# Treatment of volumetric muscle loss in mice using nanofibrillar scaffolds enhances vascular organization and integration

**DOI:** 10.1038/s42003-019-0416-4

**Published:** 2019-05-07

**Authors:** Karina H. Nakayama, Marco Quarta, Patrick Paine, Cynthia Alcazar, Ioannis Karakikes, Victor Garcia, Oscar J. Abilez, Nicholas S. Calvo, Chelsey S. Simmons, Thomas A. Rando, Ngan F. Huang

**Affiliations:** 1 Veterans Affairs Palo Alto Health Care System, Palo Alto, CA 94304 USA; 2 The Stanford Cardiovascular Institute, Stanford University, Stanford, CA 94305 USA; 3 Department of Cardiothoracic Surgery, Stanford University, Stanford, CA 94305 USA; 4 Department of Neurology and Neurological Sciences, Stanford University, Stanford, CA 94304 USA; 5 Department of Mechanical and Aerospace Engineering, University of Florida, Gainsville, FL 32611 USA

**Keywords:** Musculoskeletal models, Tissues, Tissue engineering, Cell therapies, Regeneration

## Abstract

Traumatic skeletal muscle injuries cause irreversible tissue damage and impaired revascularization. Engineered muscle is promising for enhancing tissue revascularization and regeneration in injured muscle. Here we fabricated engineered skeletal muscle composed of myotubes interspersed with vascular endothelial cells using spatially patterned scaffolds that induce aligned cellular organization, and then assessed their therapeutic benefit for treatment of murine volumetric muscle loss. Murine skeletal myoblasts co-cultured with endothelial cells in aligned nanofibrillar scaffolds form endothelialized and aligned muscle with longer myotubes, more synchronized contractility, and more abundant secretion of angiogenic cytokines, compared to endothelialized engineered muscle formed from randomly-oriented scaffolds. Treatment of traumatically injured muscle with endothelialized and aligned skeletal muscle promotes the formation of highly organized myofibers and microvasculature, along with greater vascular perfusion, compared to treatment of muscle derived from randomly-oriented scaffolds. This work demonstrates the potential of endothelialized and aligned engineered skeletal muscle to promote vascular regeneration following transplantation.

## Abstract (web summary)

Karina Nakayama et al. bioengineer endothelialized mouse skeletal muscle using parallel-aligned nanofibrillar scaffolds. They find that muscle produced using the aligned scaffolds developed a spatially patterned structure and showed improved endothelial interaction compared to muscle engineered with randomly-oriented scaffolds.

## Introduction

Skeletal muscle plays an important role in the human body for movement, mastication, and other dynamic actions. In the presence of severe muscular injuries, current treatment approaches are limited to grafting of autologous muscle flaps or scar tissue debridement^1–3^. However, these therapies are often associated with donor site morbidity due to impaired endogenous regeneration and revascularization capacity. Engineered muscle grafts are a promising alternative source for muscle flaps, but long-term tissue survival depends on rapid revascularization to meet the high metabolic demands of muscle.

As a highly structurally organized tissue, skeletal muscle is physiologically composed of bundles of parallel-aligned myofibers, interspersed with blood vessels that are also organized generally in parallel^4,5^. Besides facilitating blood flow to muscle under physiological conditions, vascular endothelial cells release angiogenic factors and promote microvasculature formation in the presence of injury^6^. Experimental approaches to induce muscle and vascular regeneration by the delivery of myogenic or angiogenic growth factors^7–11^ or other biomolecules^12,13^ have shown therapeutic potential in preclinical models of muscle injury^7,12^. Other experimental approaches such as the transplantation of therapeutic cells or minced tissues^14–19^ are limited by the ability to control the structural organization of newly formed muscle and associated vasculature. Full restoration of muscle regeneration likely requires the participation of multiple factors and cell types^20^.

To overcome these limitations, a number of bioengineering techniques have been utilized to create spatially patterned cells and engineered tissues, including soft lithography^21^, electrospinning^22^, molding^23^, three-dimensional (3D) bioprinting^24^, and shear-based extrusion^25^. In particular, shear-based extrusion of collagen takes advantage of the pH dependency of collagen fibrillogenesis, in which shearing of acidic monomeric collagen into a neutral buffer induces fibrillogenesis along the direction of extrusion to create strip-like scaffolds composed of aligned nanofibrils^26^. Unlike other approaches, shear-based collagen extrusion does not rely on specialized equipment or toxic solvents and can be used to engineer long-and-thin scaffolds that resemble the geometry of muscle fibers. When the strip-like scaffolds are aggregated in parallel, the scaffolds assemble into 3D structures that mimic the organization of muscle bundles. We have previously shown that cells seeded on aligned nanofibrillar scaffolds promote cytoskeletal organization along the direction of nanofibrillar alignment^27–29^. These studies highlight the utility of spatial patterning in generating highly ordered tissues such as skeletal muscle and vessels.

As muscle is a highly metabolic tissue that requires a constant source of nutrients and oxygen^30^, efforts to create vascularized muscle tissues by co-culture of myogenic cells with vascular lineages have been shown to promote in vivo vascular integration of engineered muscle constructs^31–33^. In agreement with these findings, our recent studies show that decellularized scaffolds containing muscle stem cells, along with endothelial cells and other support cells, enabled higher force production in ablated mouse muscle, compared with treatment with scaffolds lacking endothelial cells^34^. However, in the absence of instructive cues from the extracellular matrix (ECM), the newly formed myofibers and vasculature may not assemble into the organized structure of native muscle and microvasculature.

Accordingly, the objective of this study was to examine the combined effect of spatially patterned engineered muscle with endothelial cell co-culture, and to demonstrate the therapeutic benefit of endothelialized and aligned engineered muscle for enhancing vascular regeneration in a preclinical model of traumatic muscle injury. We engineered endothelialized and aligned skeletal muscle in vitro that formed highly aligned myotubes, interspersed with vascular endothelial cells. Implantation of the endothelialized aligned engineered skeletal muscle into a murine volumetric muscle loss injury model results in the formation of highly organized myofibers and microvasculature, with functional improvement in vascular perfusion and cellular retention, compared with treatment with endothelialized engineered muscle derived from randomly oriented scaffolds. Therefore, these findings may have profound implications in the design of engineered muscle that promotes vascular regeneration and organized vasculature formation following transplantation.

## Results

### Organization of endothelialized engineered skeletal muscle

**Fig. 1:**
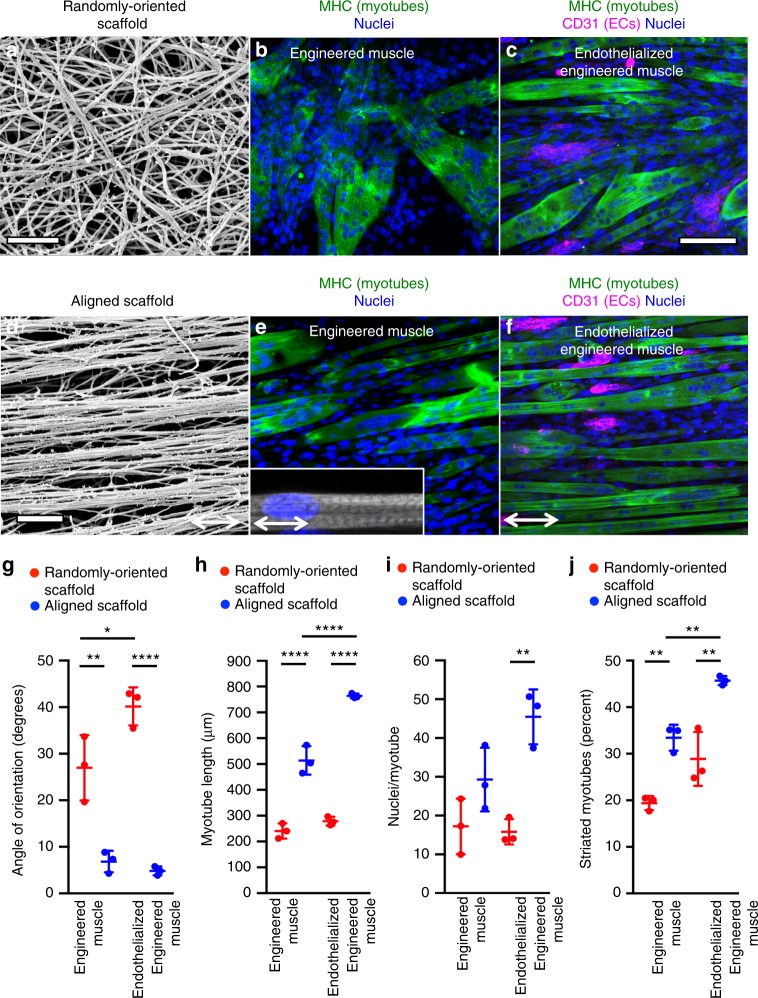
In vitro characterization of endothelialized engineered murine muscle. Engineered skeletal muscle was formed by the fusion of murine myoblasts within randomly oriented or aligned nanofibrillar scaffolds for 9 days. Co-culture of myoblasts with endothelial cells on the scaffolds formed endothelialized engineered skeletal muscle. **a**, **d** Scanning electron microscopy images of randomly oriented (**a**) or aligned (**d**) scaffold nanofibers. **b**, **c**) Confocal microscopy images depict myosin heavy chain (MHC, green) and CD31 (magenta) staining in differentiated myotubes (**b**) or myotubes co-cultured with endothelial cells (**c**) in randomly oriented scaffolds. **e**, **f** Confocal microscopy images of MHC (green) and CD31 (magenta) of differentiated myotubes (**e**) or myotubes co-cultured with endothelial cells (**f**) on aligned scaffolds. **g** Average angle of orientation of differentiated myotubes, relative to the axis of aligned nanofibrils for aligned scaffolds or an arbitrary axis for randomly oriented scaffolds (*n* = 3 each group). **h** Average myotube length on either randomly oriented or aligned scaffolds cultured in the presence or absence of endothelial cells (*n* = 3 each group). **i** Myotube fusion efficiency is expressed as the average number of nuclei per myotube (*n* = 3 each group). **j** Percentage of mature striated MHC^+^ myotubes out of total MHC^+^ myotubes (*n* = 3 each group). For indicated comparisons: *p* ≤ 0.05 (*), *p* ≤ 0.01 (**), *p* ≤ 0.0001 (****). Error bars represent standard deviation. Arrow designates orientation of aligned nanofibrillar scaffold. Scale bars denote 1 μm (**a**, **d**); 100 μm (**b**, **c**)

Using a facile shear-based extrusion method, we developed parallel-aligned nanofibrillar scaffolds by taking advantage of the pH dependency of collagen fibrillogenesis^26,35^. Extrusion of acidic collagen monomer into a pH neutral buffer in the presence of shear stress preferentially organized the nanofibrils in the direction of extrusion to create long-and-thin scaffold strips (0.5 mm thickness × 22 mm length) having parallel-aligned nanofibrillar structure^28^. The nanotopography of the scaffold was verified by scanning electron microscopy that confirmed the spatial organization and uniformity of aligned nanofibrils (30–50 nm diameter) along a uniaxial direction, whereas randomly oriented collagen nanofibrils lacked any preferential fibril orientation (Fig. 1a, d).

Murine C2C12 myoblasts were cultured on long-and-thin strips of randomly oriented or aligned nanofibrillar scaffolds, and then induced for 5 days to form multi-nucleated myotubes by the reduction of serum nutrients. Afterward, human microvascular endothelial cells were introduced into the scaffolds for an additional 4 days (Supplementary Fig. 1). Myoblasts and endothelial cells were fluorescently labeled using green fluorescence protein (GFP) and mRuby/mCherry, respectively, to distinguish between cell types (Supplementary Fig. 2). To assess the organization of the myotubes that formed in the aligned scaffolds, myotube orientation and length were evaluated after 9 days. Based on immunofluorescence staining of myosin heavy chain (MHC), the myotubes in engineered skeletal muscle formed from aligned scaffolds were highly organized along the direction of the nanofibrils (Fig. 1e, f). The degree of alignment was quantified in which an orientation perfectly parallel to the nanofibers corresponded to an angle of 0°, whereas myotubes organized perpendicular to the axis of the nanofibrils would have an angle of 90°. The myotubes in aligned scaffolds had an average angle of orientation of 6.9 ± 2.3° (myoblasts only), which was similar to the alignment in the endothelialized skeletal muscle (myoblasts and endothelial cells; 4.9 ± 0.9°), suggesting that endothelialization of the aligned scaffolds did not interfere with myotube alignment (Fig. 1e–g). In contrast, myotubes of the engineered muscle formed from randomly oriented scaffolds were more disorganized, as reflected by a significantly larger angle of orientation (27.0 ± 7.0°, *p* ≤ 0.01). Endothelialized engineered muscle formed from randomly oriented scaffolds also had a significantly larger angle of orientation (40.2 ± 4.1°, *p* ≤ 0.0001), compared with corresponding endothelialized engineered muscle formed from aligned scaffolds (Fig. 1b, c, g).

Besides inducing organized myotube assembly, the aligned nanofibrillar scaffolds also promoted the formation of significantly longer myotubes (Fig. 1h). Myotubes within engineered skeletal muscle formed from aligned scaffolds had an average length of 510 ± 60 µm, and corresponded to 760 ± 10 µm for endothelialized skeletal muscle. In contrast, engineered muscle in randomly oriented scaffolds had significantly shorter lengths (240 ± 30 µm), with a corresponding length of 280 ± 20 µm in endothelialized engineered muscle (*p* ≤ 0.0001, Fig. 1h). The myotube length was significantly longer in aligned scaffolds in the presence of endothelial cells, compared with in the absence of endothelial cells (*p* ≤ 0.0001), suggesting that spatially patterned endothelial cells were beneficial for myotube formation. Additional quantification of myotube size based on the number of nuclei per myotube showed similar findings in which myotubes in aligned scaffolds had significantly more nuclei per myotube, compared with that in randomly oriented scaffolds (*p* ≤ 0.01, Fig. 1i).

To evaluate the quality of mature myotubes, the percentage of myotubes showing a striated MHC expression pattern^27^ was assessed and found to be significantly higher in endothelialized engineered skeletal muscle formed from aligned scaffolds (45 ± 1%), compared with that in randomly oriented scaffolds (28 ± 4%; *p* ≤ 0.01), or skeletal muscle formed from aligned scaffolds (34 ± 2%; *p* ≤ 0.01) (Fig. 1j). These results indicate that, in comparison with randomly oriented scaffolds, aligned nanofibrillar scaffolds induced parallel alignment of myotubes, longer myotubes, and a higher percentage of striated myotubes. Importantly, endothelialization of the engineered muscle further promoted an increase in myotube length and the percentage of striated myotubes in aligned nanofibrillar scaffolds.

### Endothelialized engineered skeletal muscle function

**Fig. 2:**
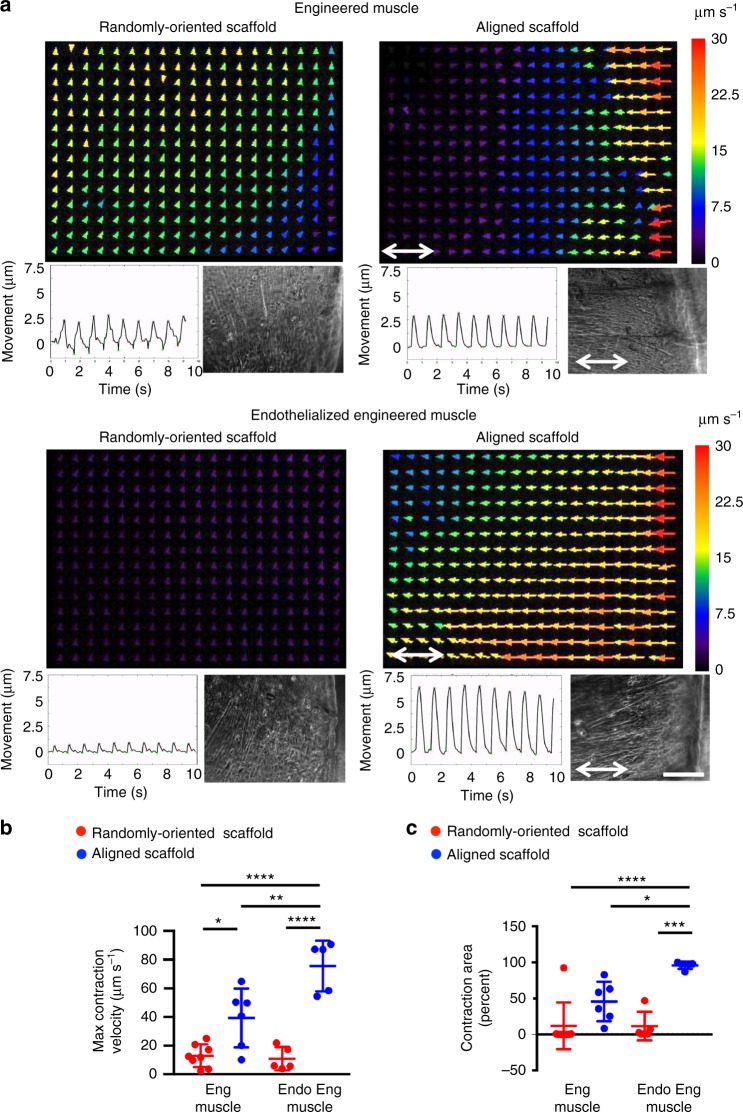
In vitro contraction of endothelialized engineered murine skeletal muscle. Engineered skeletal muscle (denoted as Eng Muscle) was formed by the fusion of myoblasts within randomly oriented or aligned nanofibrillar scaffolds for 9 days. Co-culture of myoblasts with endothelial cells on the scaffolds formed endothelialized engineered skeletal muscle (denoted as Endo Eng Muscle). **a** Colored vector plots show the magnitude and direction of representative electrically paced constructs after 9 days of culture. Shown below the colored vector plots are global movement (μm) as a function of time (s) and bright field images of engineered muscle. Scale bar denotes 500 μm. **b** Maximum contraction velocity (μm s^–1^) and (**c**) percentage of contracting area for engineered skeletal muscle, with or without endothelialization, formed from randomly oriented or aligned scaffolds (*n* ≥ 5). For indicated comparisons: *p* ≤ 0.05 (*), *p* ≤ 0.01 (**), *p* ≤ 0.001 (***), *p* ≤ 0.0001 (****). Error bars represent standard deviation. Arrow designates orientation of aligned nanofibrillar scaffold

Besides morphological assessment of myoblasts in aligned nanofibrillar scaffolds, the contractile function in response to electrical stimulation was assessed on day 9 after cell seeding. Global contractile properties were quantified by video recordings of electrically paced (1 Hz) engineered skeletal muscle constructs. Using the particle image velocimetry plugin in ImageJ and a custom MATLAB script^36^, the magnitude and direction of electrically paced tissues were depicted as colored vector plots (Fig. 2a). Endothelialized engineered muscle formed from aligned scaffolds demonstrated greater contractile magnitudes (colored vector plots) and more highly synchronized movement, compared with engineered muscle formed from randomly oriented scaffolds (Supplementary Movies 1, 2). Endothelialized engineered muscle formed from aligned scaffolds also showed over a six-fold increase in maximum contraction velocity (*p* < 0.0001), as well as over an eightfold increase in contracting area (*p* < 0.001), when compared with those formed from randomly oriented scaffolds (Fig. 2b, c). These data suggest that endothelialized engineered muscle formed from aligned scaffolds were highly responsive to electrical stimulation and displayed uniform and robust contractile function.

### Cytokine production by endothelialized engineered muscle

**Fig. 3:**
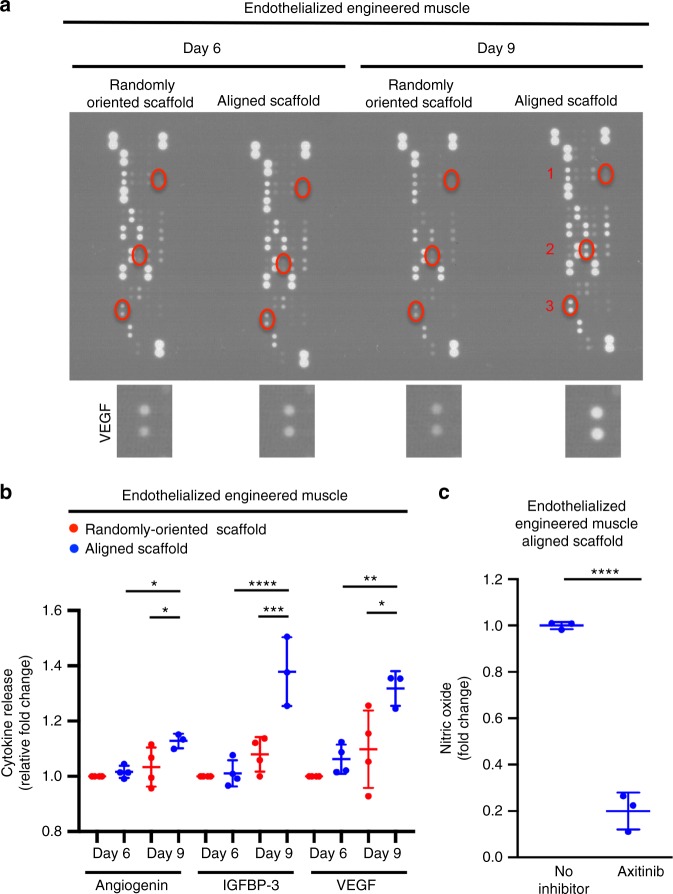
Secretion of angiogenic and myogenic cytokines by endothelial cells in endothelialized engineered skeletal muscle in vitro. **a** Representative proteomic array depicts the protein expression of differentially expressed cytokines secreted by endothelial cells when co-seeded with myoblasts in randomly oriented or aligned scaffolds for 6 or 9 days of culture. Highlighted in red are (1) angiogenin, (2) insulin-like growth factor binding protein-3 (IGFBP-3), and (3) vascular endothelial growth factor (VEGF) in replicate samples. Inset shows representative replicate spots depicting secreted VEGF levels. **b** Relative fold change in the protein expression of angiogenin, IGFBP-3, and VEGF compared with randomly oriented constructs after 6 days of culture (*n* ≥ 3 each group). **c** Fold change difference in nitric oxide based on the fluorescence intensity of nitric oxide fluorescent probe in the presence of axitinib, a small molecule inhibitor of VEGF receptor. Samples consist of endothelial cells co-cultured with myoblasts in randomly oriented and aligned skeletal muscle constructs for 6 days of culture (*n* ≥ 3 each group). For indicated comparisons: *p* ≤ 0.05 (*), *p* ≤ 0.01 (**), *p* ≤ 0.001 (***), *p* ≤ 0.0001 (****). Error bars represent standard deviation

To determine the mechanism by which nanofibril alignment modulates endothelial function, we performed proteomic profiling of cytokines in cell culture supernatants collected from endothelialized skeletal muscle formed from randomly oriented or aligned scaffolds on day 6 (1 day after introduction of endothelial cells) and day 9 (4 days after introduction of endothelial cells) of culture (Fig. 3a). Owing to the human specificity of the array, we could identify cytokines released by the human endothelial cell line. In comparison with randomly oriented scaffolds on day 9, endothelialized engineered muscle formed from the aligned nanofibrillar scaffolds produced a significantly higher level of angiogenin (*p* ≤ 0.05), a cytokine that is a known stimulator of new blood vessel formation and functions by promoting endothelial cell migration, invasion, proliferation, and formation of tubular structures (Fig. 3b). Similarly, on day 9, endothelialized engineered muscle formed from aligned scaffolds produced a significantly higher level of vascular endothelial growth factor alpha (VEGF-A, *p* ≤ 0.05), a cytokine that plays a major role in angiogenesis by promoting proliferation and migration of vascular endothelial cells. In addition, the dually myogenic and angiogenic factor insulin-like growth factor binding protein-3 (IGFBP-3) was also significantly elevated in endothelialized engineered muscle formed from aligned scaffolds (*p* ≤ 0.001 on day 9). In comparison with day 6, there was an increase in the relative levels of these cytokines on day 9, presumably due to the longer period of intercellular interaction between endothelial cells and myotubes in the skeletal muscle tissue constructs.

To validate the role of VEGF-A in modulating endothelial function within the endothelialized engineered muscle, pharmacological inhibition of VEGF-A was performed using axitinib, a small molecule inhibitor of VEGF receptor signaling^37,38^. In the presence of axitinib, endothelial cells within the endothelialized engineered muscle formed from aligned scaffolds showed an 80% reduction in the production of nitric oxide (NO) (*p* ≤ 0.0001), based on the intensity of a fluorescent NO probe, as a measure of angiogenesis (Fig. 3c). These findings were further confirmed using a second VEGFR tyrosine kinase inhibitor (Supplementary Fig. 3). Together, these data demonstrate increased secretion of angiogenic and myogenic cytokines by endothelial cells in engineered skeletal muscle formed from aligned scaffolds, compared with randomly oriented scaffolds. These data care consistent with alignment-mediated modulation of endothelial cells and enhancement of angiogenic phenotype.

### RNA sequencing of endothelialized engineered skeletal muscle

**Fig. 4:**
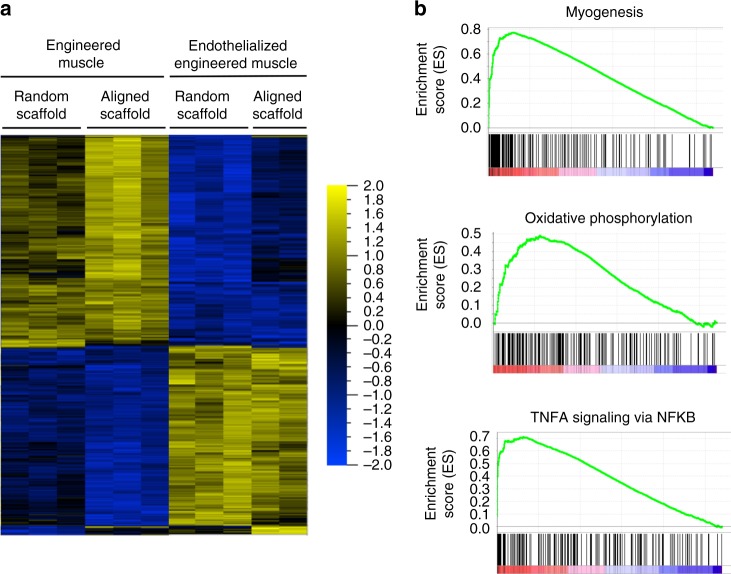
Global gene expression profiles of endothelialized engineered muscle derived from randomly oriented or aligned scaffolds. **a** Heat map of the top 500 differentially expressed genes by engineered muscle or endothelialized engineered muscle grown in randomly oriented (random) or aligned scaffolds (false discovery rate *q*-value < 0.05). **b** Gene Set Enrichment Analysis shows selected gene ontology data sets enriched in the endothelialized engineered muscle derived from aligned scaffolds, in comparison with randomly oriented scaffolds. Gene ontology data sets are shown for myogenesis, oxidative phosphorylation, and tumor necrosis factor-α (TNFA) via nuclear factor κ-light-chain-enhancer of activated B cells (NKFB)

To gain further insights into the molecular mechanisms by which spatial patterning promoted myogenesis and angiogenesis, we performed mRNA sequencing of engineered muscle and endothelialized engineered muscle on either randomly oriented or aligned scaffolds (Fig. 4a). Among the differentially expressed genes, 438 were significantly upregulated and 423 were downregulated in engineered muscle formed from aligned scaffolds, compared with randomly oriented scaffolds (*q* ≤ 0.05). Among the significantly upregulated genes included angiogenic factors like *VEGFA*, fibroblast growth factor receptor-3 (*FGFR3*), and fibroblast growth factor-11 (*FGF11*). Similarly, 95 genes were differentially regulated between endothelialized engineered muscle formed from aligned scaffolds, compared with randomly oriented scaffolds, including 89 significantly upregulated and 6 downregulated genes (*q* ≤ 0.05). Gene set enrichment analysis (GSEA) using the gene sets in the Hallmark collection^39^ revealed multiple pathways that were upregulated in endothelialized engineered muscle formed from aligned scaffolds, compared with randomly oriented scaffolds. One of the pathways was myogenesis, which was driven by genes in the MHC family such as *Myh7*, *Myh4*, *Myh2*, and *Myh1* (Fig. 4b). Similar findings of significantly upregulated myogenic genes were also found in engineered muscle formed from non-endothelialized aligned scaffolds, compared with randomly oriented scaffolds (Supplementary Fig. 4). Another notable enriched pathway was oxidative phosphorylation, which is commonly associated with increased myogenesis. For example, mitofusin 2 (*Mfn2*), which is involved in mitochondrial biogenesis in which cells increase their mitochondrial mass^40^, was significantly upregulated in endothelialized engineered muscle constructs derived from aligned scaffolds. Biogenesis is responsible for increased glucose uptake in muscle and decreased expression of the proteins associated with mitochondrial biogenesis is seen with aging and cardiovascular disease^41^. In addition, inflammation-related genes such as JunB proto-oncogene (JUNB), a transcriptional regulator of vascular development and neurovascular parallel alignment^42^, and a family of Fos genes (*Fos, Fosb, Fosl2,* and *Fosl1*) were upregulated on endothelialized engineered muscle constructs on aligned scaffolds when compared with randomly aligned scaffolds. These two gene families complex with activator protein-1 (AP-1) in a VEGF-induced endothelial activation cascade known to promote cell migration and angiogenesis^43^. Together, these findings suggest that spatial patterning of the scaffolds induce differential transcriptional pathways that may regulate engineered muscle behavior.

### Implantation of engineered skeletal muscle in injured muscle

**Fig. 5:**
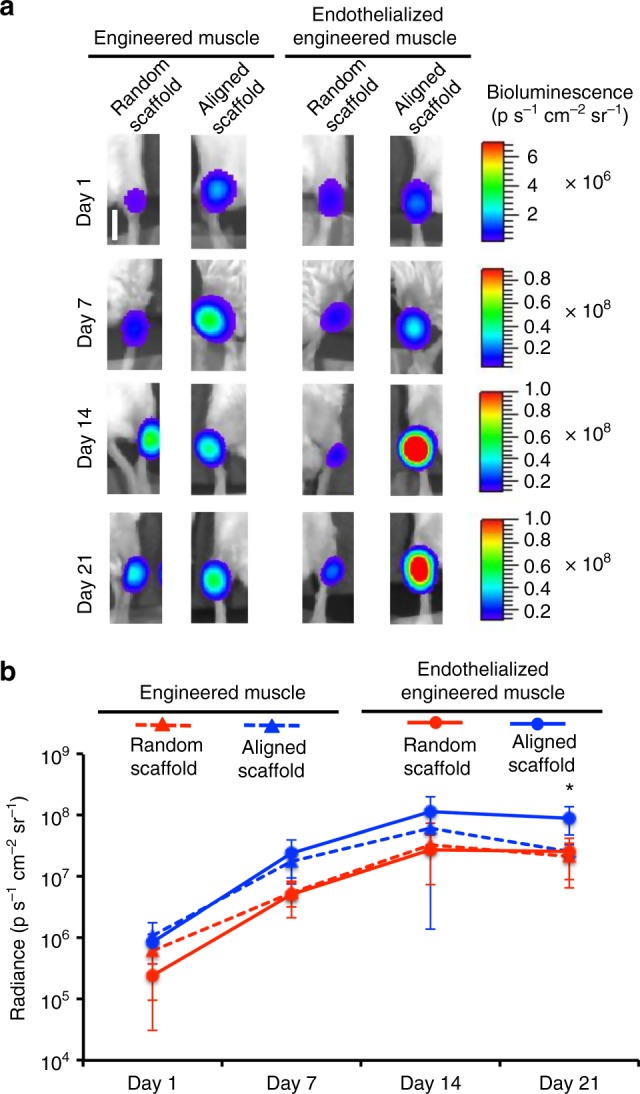
Bioluminescence imaging of donor mouse myoblast survival in a murine model of volumetric muscle loss. **a** Representative bioluminescence images of bioluminescently labeled myoblasts within endothelialized engineered skeletal muscle formed from randomly oriented or aligned scaffolds. **b** Quantitative analysis is shown of the bioluminescence signal on days 1, 7, 14, and 21 days after transplantation into the ablated muscle of mice (*p ≤ 0.05) compared with all other groups (*n* = 4). Error bars represent standard deviation. Scale bar denotes 1 cm

To evaluate the therapeutic efficacy of endothelialized skeletal muscle constructs, traumatic muscle injury was induced by surgical removal of 20% of the tibialis anterior muscle in NOD SCID mice, creating a defect with limited endogenous regenerative capacity and functional impairment^34,44^. The engineered skeletal muscle constructs were transplanted into the ablated muscle (Supplementary Fig. 5). To generate 3D tissues for transplantation, a bundle of eight aligned nanofibrillar scaffolds (9 mm × 2 mm × 3 mm) was seeded with mouse myoblasts co-seeded with or without endothelial cells, forming aligned skeletal muscle bundle with or without endothelialization (Supplementary Fig. 6). Similarly, a bundle of eight randomly oriented nanofibrillar scaffolds was also seeded with myoblasts with or without endothelial cells, forming randomly oriented skeletal muscle constructs with or without endothelialization. An acellular aligned scaffold-only negative control was also transplanted (Supplementary Fig. 6). To track the survival of myoblasts in vivo, the cells were genetically modified to dually express GFP and firefly luciferase under a constitutively active promoter^45^. Survival of the myoblast population was monitored non-invasively by bioluminescence imaging, in which bioluminescence intensity correlates linearly to viable cell numbers^46^. The trajectory of the bioluminescent signal in all cell-seeded groups demonstrated a proliferative phase between days 1 and 14, followed by a phase of self-stabilization between days 14 and 21 (Fig. 5a, b). Although all cell-seeded groups increase in bioluminescence over time, the animals treated with aligned endothelialized skeletal muscle tissue constructs showed significantly greater bioluminescence intensity after 21 days (*p* ≤ 0.05), in comparison with all other groups (Fig. 5b).

**Fig. 6:**
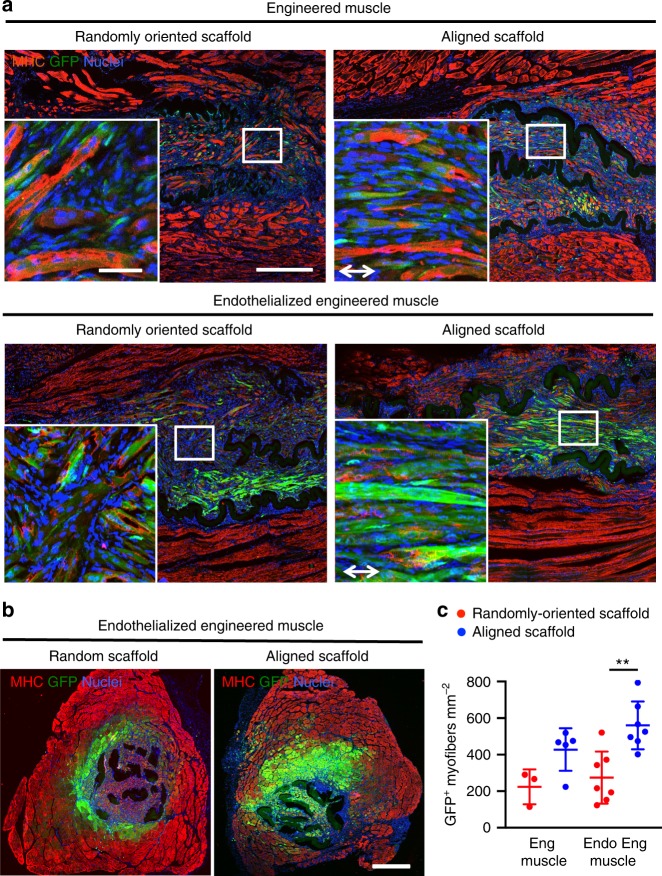
Implantation of murine engineered skeletal muscle in a mouse model of volumetric muscle loss. **a** Longitudinal sections of endothelialized engineered muscle formed randomly oriented or aligned skeletal muscle constructs. Donor transplanted myofibers are denoted by the co-expression of green fluorescence protein (GFP, green) and myosin heavy chain (MHC, red). Native myofibers are denoted by MHC^+^/GFP^-^ expression. Insets show higher magnification images of the GFP^+^ region depicted by white boxes in the center of the engineered tissues. **b** Representative image of a tissue cross-section of muscle transplanted with an endothelialized engineered skeletal muscle construct at 21 days after induction of traumatic muscle injury. MHC (red) indicates mature myofibers, GFP (green) indicates the transplanted myoblast population, and nuclei are visualized in blue using Hoechst 33342 dye. **c** The density of MHC^+^/GFP^+^ transplanted myofibers in the region of regeneration is shown (*n* ≥ 3, **p ≤ 0.01). Error bars represent standard deviation. Arrow designates orientation of aligned nanofibrillar scaffold. Scale bars denote 50 μm (inset) or 500 μm (**a**); 500 μm (**b**)

The formation of donor-derived myofibers was examined histologically in explanted tibialis anterior muscles at 21 days after implantation. We observed partial degradation of the scaffolds from the Trichrome staining of muscle tissue cross-sections used to visualize the collagen scaffolds (Supplementary Fig. 7). Hematoxylin and eosin staining showed scaffold integration into the defect site with minimal scar formation (Supplementary Fig. 7). Acellular scaffold-only transplants provided supportive structure that filled the void space of the muscle defect, but de novo myofiber formation and endogenous muscle regeneration was minimal (Supplementary Fig. 7). In contrast, engineered muscle formed from both randomly oriented and aligned scaffolds demonstrated extensive muscle fiber formation originating from donor-derived cells, which were characterized by the co-expression of GFP and MHC (Fig. 6a and Supplementary Figs. 7, 8). In animals treated with engineered muscle formed from aligned scaffolds, the donor-derived myofibers constituted a large region of regenerated tissue that extended several hundred microns beyond the boundaries of the scaffold (Fig. 6b). In comparing the endothelialized treatment groups, animals treated with endothelialized engineered skeletal muscle in aligned scaffolds led to twice as many donor-derived regenerating myofibers than in randomly oriented scaffolds (Fig. 6c, *p* ≤ 0.01).

To determine whether the newly regenerated muscle fibers retained aligned muscle organization, muscle tissues were sectioned longitudinally, relative to the orientation native muscle fibers. Longitudinal alignment of the transplanted myofibers was most pronounced in the endothelialized engineered skeletal muscle formed from aligned scaffolds, in which the donor-derived myofibers were organized in parallel with endogenous myofibers (Fig. 6a). At the distal interface between the host muscle and the endothelialized engineered skeletal muscle formed from aligned scaffolds, the donor-derived myofibers could be visualized in close proximity to native myofibers that lack GFP expression. In contrast, animals treated with acellular scaffolds or engineered muscle without endothelialization showed relatively disorganized native or donor-derived myofibers, respectively (Supplementary Figs. 8, 9). Implantation of endothelialized engineered skeletal muscle formed from randomly oriented scaffolds resulted in scattered regions of localized myofiber alignment (Fig. 6a). Together these data show a greater density of transplanted myofibers, as well as global organization along the direction of native myofibers, when transplanted with endothelialized engineered skeletal muscle formed from aligned scaffolds, compared with randomly oriented scaffolds.

### Endothelialized muscle promotes vascular regeneration

**Fig. 7:**
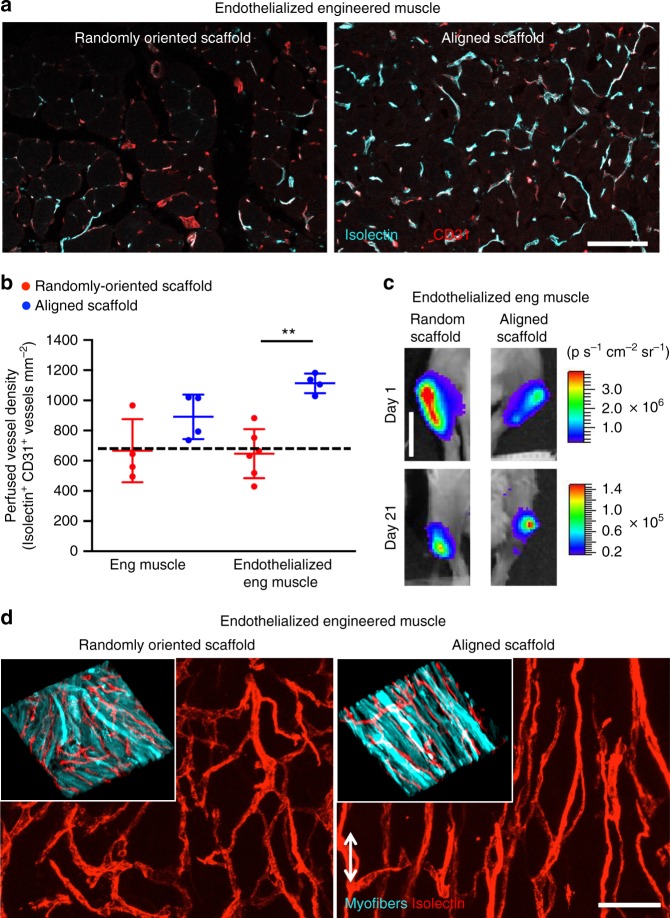
Murine endothelialized engineered skeletal muscle promotes vascular anastomosis and revascularization in a mouse model of volumetric muscle loss. **a** Representative confocal microscopy images of perfused vasculature from transverse tissue sections in the region of regeneration adjacent to the endothelialized randomly oriented or endothelialized aligned transplanted skeletal muscle constructs at 21 days after implantation. Endothelial cells are identified by CD31 (red) expression. Perfused vessels are indicated by co-expression of CD31 (red) and fluorescently labeled isolectin (turquois). **b** Perfused vessel density in the region of regeneration (*n* ≥ 4, **p ≤ 0.01). Dotted line denotes the levels of the acellular scaffold control. **c** Bioluminescence imaging of randomly oriented or aligned skeletal muscle constructs seeded with bioluminescent endothelial cells transplanted into ablated muscle. **d** Perfused vessel organization within transplanted endothelialized engineered muscle formed from randomly oriented or aligned scaffolds. Inset shows the arrangement of myofibers and the associated vessel architecture as visualized by green fluorescence protein (GFP) expression (turquois) in the transplanted myofibers and fluorescently labeled isolectin (red). Arrow designates orientation of aligned nanofibrillar scaffold. Scale bars denote 100 μm (**a**), 1 cm (**c**), and 50 μm (**d**). Error bars represent standard deviation

The high metabolic needs of native skeletal muscle requires the association of several capillaries with each myofiber^47^. Restoration of tissue vasculature is a necessary and critical component for supporting muscle regeneration, long-term recovery, and maintenance. Therefore, the extent of vascularization within and surrounding the transplanted engineered muscle was examined in transverse tissue sections. Prior to euthanasia, the mouse vasculature was perfused with fluorescently conjugated isolectin, which specifically binds to endothelial cells in perfused blood vessels (Supplementary Figs. 8, 10). Perfused vessels were identified by the co-staining of endothelial marker, CD31, and isolectin (Fig. 7a). A significantly higher density of CD31^+^/isolectin^+^ perfused vessels were found for endothelialized engineered muscle formed from aligned scaffolds (1113 ± 65 mm^−2^), which was 58% higher compared with that of randomly oriented scaffolds (647 ± 162 mm^−2^, *p* ≤ 0.01) (Fig. 7b). Moreover, the endothelialized engineered skeletal muscle derived from aligned scaffolds induced significantly higher perfused vascular density, when compared with the acellular transplanted scaffolds (676 ± 107 mm^−2^, *p* ≤ 0.01). The transplanted endothelial cells remained viable during the course of the 21 days, based on bioluminescence imaging of bioluminescent endothelial cells (Fig. 7c). Together, these data suggested that treatment of endothelialized engineered muscle formed from aligned scaffolds supported a higher degree of vascular perfusion, in comparison with treatment of muscle derived from randomly oriented scaffolds.

### Endothelialized muscle promotes organized microvasculature

**Fig. 8:**
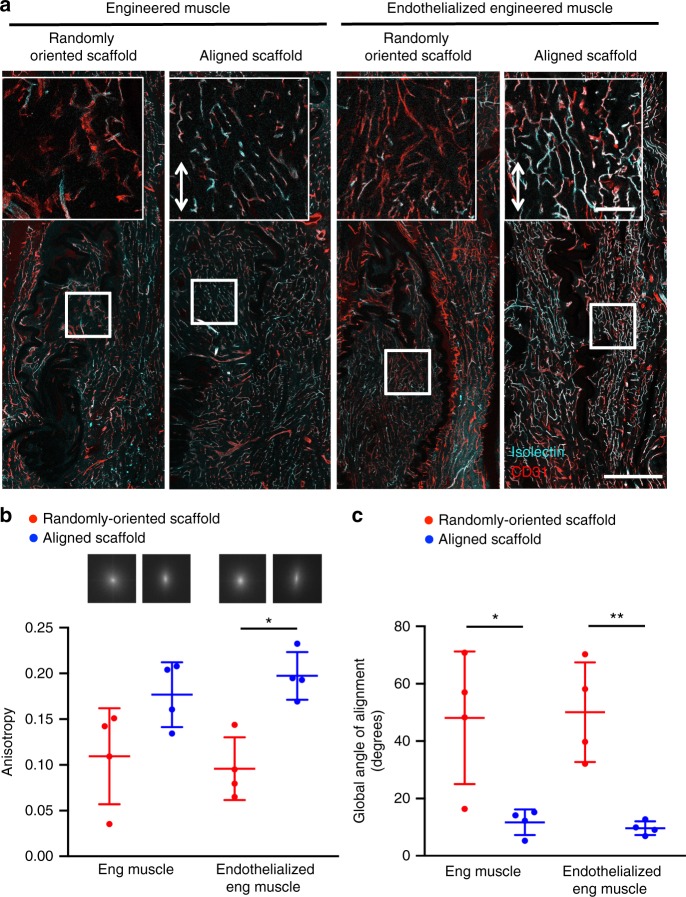
Murine endothelialized engineered skeletal muscle supports the formation of organized vascular networks in traumatically injured muscle. **a** Representative confocal microscopy images of perfused vasculature from longitudinal tissue sections in the region of regeneration adjacent to the endothelialized randomly oriented or endothelialized aligned transplanted skeletal muscle constructs at 21 days after implantation. Endothelial cells are identified by CD31 (red) expression. Perfused vessels are indicated by co-expression of CD31 (red) and fluorescently labeled isolectin (turquois). Insets are higher magnification images of the center of the engineered tissues as depicted by white boxes. Arrow designates orientation of aligned nanofibrillar scaffold. Scale bar (inset) denotes 100 μm, Scale bar denotes 500 μm. **b** Anisotropy is used to quantify the directional dependence towards the formation of parallel-aligned vascular networks. A value approaching zero corresponds to unrestricted (non-patterned) paths, whereas a value approaching one denotes restriction to one axis to form a parallel-aligned spatial pattern (*n* = 4). Inset shows representative frequency plots generated by two-dimensional Fast Fourier Transform analysis, in which random orientation of vascular networks are depicted as pixels evenly distributed about the central origin, whereas parallel alignment of vascular networks is depicted as pixels preferentially organized along a single axes (vertical). Quantification of engineered muscle (Eng Muscle) and endothelialized engineered muscle (Endothelialized Eng Muscle) treatment groups is shown. **c** Global angle of alignment of vascular networks was quantified as the angle formed by the relative orientation of the vascular networks, relative to the axis of the aligned nanofibrils. A degree of alignment of 0 depicts parallel alignment of vascular networks to the aligned nanofibrils. For randomly oriented scaffolds, an arbitrary axis is used (*n* = 4). For indicated comparisons: *p* ≤ 0.05 (*), *p* ≤ 0.01 (**). Error bars represent standard deviation

As native vasculature in fast twitch skeletal muscle, such as the TA, is highly aligned at both the tissue and the sub-tissue level^47^, we further assessed whether the spatial patterning of the scaffold could modulate the vascular organization. Isolectin-perfused vessels surrounding the donor-derived GFP-expressing myofibers were abundant, demonstrating extensive vascular integration with the host vasculature, based on analysis of longitudinal tissue sections. The perfused vessels surrounding the endothelialized engineered muscle formed from randomly oriented scaffolds were shorter in length and lacked order (Fig. 7d). In contrast, the vessels surrounding the endothelialized engineered muscle formed from aligned scaffolds were highly organized in parallel with the GFP^+^ myofibers and closely resembled the organization of the native vasculature (Fig. 7d). To quantify the degree of organization of newly formed vessels, architectural characteristics were measured in longitudinal tissue sections of 50 μm thickness (Fig. 8a). Based on the CD31 expression of the vasculature, the global longitudinal architecture of newly formed blood vessels adjacent to the muscle transplants demonstrated marked differences in fractional anisotropy, a measure of directional dependence in which a value approaching zero corresponds to unrestricted (non-patterned) paths and a value approaching one denotes restriction to one axis (parallel-aligned spatial pattern). Vessels formed from endothelialized engineered muscle and aligned scaffolds had greater anisotropy (0.197 ± 0.026) compared with randomly oriented scaffolds (0.096 ± 0.034), suggesting greater spatial patterning along the axis of the nanofibrillar scaffold (Fig. 8b, *p* ≤ 0.05). Furthermore, analysis of the vessels within the muscle implant demonstrated a four-fold lower global angle of orientation when implanted with engineered muscle (12 ± 4°, *p* ≤ 0.05) or endothelialized engineered muscle (10 ± 2°, *p* ≤ 0.01) derived from aligned scaffolds, compared with that derived from randomly oriented scaffolds (48 ± 23° and 50 ± 17°, respectively), indicating a high degree of parallel alignment of newly formed vasculature along the direction of the tissue myofibers (Fig. 8c). These results suggested that aligned nanofibrillar scaffolds promoted the patterned organization of microvasculature, which better resembled physiological microvascular architecture within muscle.

### Engineered muscle comprising primary human cells

**Fig. 9:**
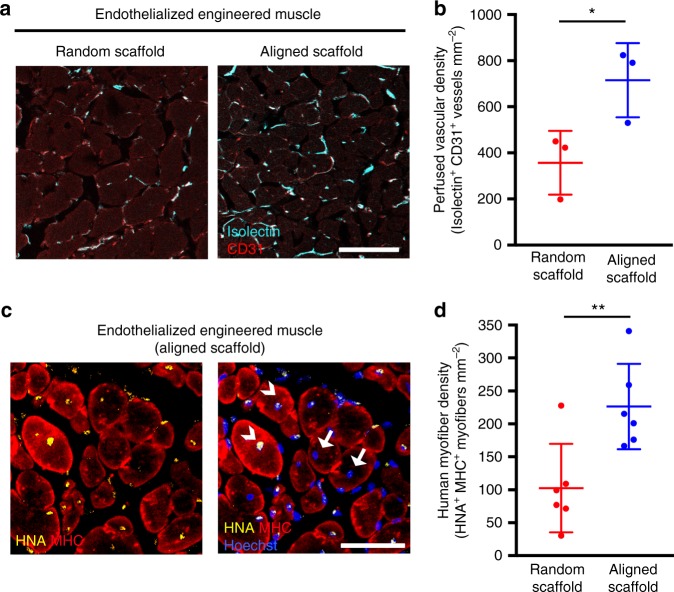
Transplantation of human endothelialized skeletal muscle into traumatically injured muscle. Human endothelialized skeletal muscle bundles were composed of primary human cells. **a** Representative confocal microscopy images of perfused vasculature in the region of regeneration adjacent to endothelialized muscle formed from randomly oriented or aligned scaffolds are shown. Endothelial cells are identified by CD31 (red) expression. Perfused vessels are indicated by co-expression of CD31 (red) and fluorescently labeled isolectin (turquois). **b** Perfused vessel density adjacent to the site of engineered muscle transplantation (*n* = 3). **c** Representative image of donor-derived myofibers in the region of regeneration adjacent to endothelialized muscle formed from randomly oriented or aligned scaffolds. Donor-derived myofibers are identified by the dual co-expression of human nuclear antigen (HNA, yellow) and myosin heavy chain (MHC, red). Arrows denote native myofibers and arrowheads denote donor-derived myofibers. **d** The density of donor-derived (HNA^+^/MHC^+^) myofibers in the region of regeneration is shown (*n* = 6). For indicated comparisons: *p* ≤ 0.05 (*), *p* ≤ 0.01 (**). Error bars represent standard deviation. Scale bars denote 100 μm (**a**); 150 μm (**c**)

To further explore the translational potential of this tissue engineering approach, endothelialized human skeletal muscle using primary muscle precursor cells (hMPCs) and primary human microvascular endothelial cells was developed (Supplementary Fig. 11a). The human muscle precursor cells expressed Pax7 at both the protein and transcriptional level (Supplementary Fig. 11b). Similar to mouse myoblasts, the human muscle precursor cells aligned their cytoskeletal bodies along the direction of the scaffold nanofibers. After 9 days of differentiation in culture, the myotubes in randomly oriented constructs had a significantly larger average angle of orientation (43.5 ± 4°), compared with that of aligned skeletal muscle constructs (4.5 ± 0.4°, *p* < 0.01, Supplementary Fig. 11c). The endothelialized engineered skeletal muscle composed of a bundle of eight cell-seeded scaffold strips were transplanted into the injured tibialis anterior muscle and histologically assessed for muscular and vascular regeneration. Perfused vessels were identified based on co-staining of CD31 and isolectin (Fig. 9a). Quantification of perfused vascular density (Fig. 9b and Supplementary Fig. 12) demonstrated that treatment of endothelialized human skeletal muscle formed from aligned scaffolds promoted >2-fold greater perfused vessel density, compared with that of randomly oriented scaffolds (*p* ≤ 0.05). Co-expression of human-specific nuclear antigen and MHC was used to identify myofibers derived from donor human muscle precursor cells (Fig. 9c). Endothelialized human skeletal muscle formed from aligned scaffolds also demonstrated a >2-fold increase in donor-derived human myofiber density, *p* ≤ 0.01), compared with that of randomly oriented scaffolds (Fig. 9d). Taken together, these data demonstrate the clinical translational potential and regenerative capacity of endothelialized skeletal muscle generated from primary human cells as a tissue engineering treatment for muscle injury or myopathies.

## Discussion

Although the intrinsic regenerative capacity of skeletal muscle is well-documented, the ability for self-repair is functionally impeded when a critical volume of muscle is damaged and normal physiological muscle regeneration is replaced with fibrotic ECM deposition and scar tissue formation. Therapeutic interventions aim to restore physiological structure and function by restoring muscle architecture and vasculature. The current study utilizes aligned nanofibrillar scaffolds and endothelial cells to overcome the challenge of restoring vascular regeneration in the setting of traumatic muscle loss. The salient findings are: (1) proteomic and transcriptional analysis of endothelialized engineered muscle derived from aligned scaffolds secreted higher levels of myogenic and angiogenic cytokines and upregulated angiogenic genes, in comparison with endothelialized engineered muscle derived from randomly oriented scaffolds (Figs. 3, 4); (2) following transplantation into a mouse model of volumetric muscle loss, the endothelialized engineered muscle formed from aligned scaffolds promoted a higher degree of vascular perfusion, compared with endothelialized engineered muscle formed from randomly oriented scaffolds, and recapitulated the aligned organization of native muscle and capillary structure for efficient integration (Figs. 7, 8); and (3) transplantation of human endothelialized engineered skeletal muscle showed similar therapeutic benefits in enhancing vascular perfusion (Fig. 9).

Unlike soft lithographic approaches to create micropatterned channels, posts, or ridges that modulate whole-cell contact guidance^48^, our anisotropic scaffolds operate through nano-scale sub-cellular interactions. Nano-scale fibrils closely mimic the aspect ratio and scale of several ECM proteins such as collagen, which exhibits a diameter on the order of a few hundred nanometers^49^. Although collagen-type I is not the predominant form of collagen in native muscle, we selected this type because of its technical ability to undergo oriented fibrillogenesis in a shear-dependent manner and give rise to nano-scale fibrils in a facile manner. Myoblasts respond to these nano-scale cues by the alignment of their actin filaments along the direction of the fibers and have been noted to increase their expression of stretch-activated channels such as the transient receptor potential cation channel-1^50^, as well as greater finely tuned regulation of adhesion scaffolding proteins such as receptor activated C kinase 1 compared with micropatterned materials^51^. Cellular filopodia are highly sensitive to nanotopographical features as tiny as 10 nm and can alter their guidance mechanisms at the single-integrin scale^52^. Therefore, the use of aligned nano-scale topography allowed us to achieve remarkably efficient and robust myoblast fusion into highly aligned, striated, and contractile myotubes in vitro.

The functionality of engineered skeletal muscle was enhanced by co-culture with endothelial cells. Previous studies have reported the synergistic benefits of co-culture of myogenic cells with supportive cell types such as fibroblasts or endothelial cells to enhance myogenesis and myotube formation^53,54^. Besides incorporating into newly formed vasculature in vivo, endothelial cells have the ability to secrete pro-angiogenic and/or pro-myogenic growth factors as a way of inducing angiogenesis. Compared with randomly oriented scaffolds, transplantation of aligned scaffolds induced the secretion of myo-angiogenic cytokines including angiogenin, IGFBP-3, and VEGF-A (Fig. 3). These factors are potent modulators of angiogenesis, as well as promoters of myoblast differentiation and fusion^55^. Of notable interest is that these restorative outcomes were highly dependent upon both endothelialization, as well as aligned nanotopographical patterning of the underlying scaffold (Figs. 5–9).

Engineered skeletal muscle has tremendous potential to restore key muscle components including the muscle myofibers, macro- and microvasculature, and supportive connective tissue. Native muscle contains large arteries that are aligned along the long axes of the tissue, parallel to muscle fascicle bundles, and capillary networks are embedded in the endomysium and travel parallel to the individual muscle myofibers^47^. This organization enables efficient oxygen and nutrient transport along the length of the tissue and is an important spatial property vital to the structural restoration of skeletal muscle tissue. Sasagawa et al. generated a vascularized skeletal muscle construct through the stacking of endothelial and myoblast cell sheets that were harvested using temperature responsive poly (*N*-isopropylacrylamide). Upon subcutaneous implantation into nude mice, the constructs underwent anastomosis with the host vasculature and enabled graft survival^56^. Enhanced angiogenesis, blood perfusion, and graft survival was achieved using a decellularized extracellular matrix scaffold combined with myoblasts, endothelial cells, and fibroblasts following transplantation into the abdominal wall of mice^31,32^ compared with constructs with myoblasts alone.

Our previous studies utilizing a cell-based tissue engineering approach highlight the importance of endothelial cells, among the other muscle resident cells, in supporting de novo muscle formation and de novo vascularization in treating traumatic muscle loss^20,34^. In the current work, we build upon our previous studies by demonstrating that spatially patterned nanofibrillar scaffolds not only guided cellular orientation but also augmented therapeutic benefit. Our findings suggest that both cellular composition and spatial organization of the engineered skeletal muscle are important determinants of therapeutic benefit. We show that the synergistic advantages of spatial patterning and endothelial interactions are, in part, due to the release of angiogenic soluble factors and activation of transcriptional pathways. Furthermore, this work enabled the decoupling of the therapeutic roles of cellular alignment and cellular composition, through the use of well-defined scaffolds and cell populations, which will aide in better understanding the underlying molecular mechanisms as well as for expediting clinical translation. However, future work is warranted to evaluate the functional benefit of endothelialized muscle and spatially patterned scaffolds in muscle force generation. Another direction is the incorporation of additional support cells within the endothelialized engineered muscle such as fibroblasts or neural progenitor cells that could improve the stability of the vasculature or innervate the engineered muscle. Future studies to evaluate intercellular and extracellular matrix interactions within the engineered muscle may reveal new insights into the underlying pathways that mediate revascularization and tissue regeneration. Broadly, the findings from this study can be applied for engineering of other anisotropic tissues (i.e., cardiac muscle), as well as to injuries or diseases that require reconstructive muscle flaps.

In summary, endothelialized engineered skeletal muscle formed from aligned scaffolds demonstrated extensive vascular perfusion and organized microvasculature in a preclinical model of traumatic muscle injury. The findings from these studies provide important insights that will inform regenerative strategies and engineered therapeutics for revascularization of severely damaged skeletal muscle tissue. Furthermore, endothelialized engineered skeletal muscle serves as an innovative platform and important step in the treatment of a broad range of musculoskeletal and vascular diseases, toward the overall goal of increasing patient mobility and quality of life.

## Methods

### Fabrication of nanofibrillar collagen scaffold strips

Generation of nanofibrillar collagen scaffold strips has been described in previous publications^26^. Briefly, rat-tail collagen-type I (10 mg ml^−1^ in 0.02 N in acetic acid, pH 3.5, Corning) was dialyzed for approximately 35 min at 4 °C to obtain a final concentration of 30 mg ml^−1^ using a semi-permeable cellulose dialysis tubing (pore size 32 × 20.4 mm, Thermo Fisher) surrounded by polyethylene glycol (Sigma). The aligned nanofibrillar collagen scaffold strip (25 mm × 1 mm) was extruded from a 22G blunt tip needle onto glass slides at a roughly 30° angle with a velocity of 340 mm s^−1^. Extrusion was performed with the needle tip submerged within 10× phosphate-buffered saline (PBS, pH 7.4) warmed to 37 °C to initiate fibrillogenesis instantaneously as the collagen is extruded. A delayed fibrilogenesis approach was used to generate randomly oriented scaffold strips of a similar size by extrusion under dry conditions followed by submersion in 10× PBS. The nanostructure and fibril alignment of the scaffolds were visualized by routine scanning electron microscopy^26^.

### Culture of mouse myoblast and human endothelial cell lines in scaffolds

Mouse myoblasts (C2C12, ATCC) were expanded in a high serum Dulbecco’s modified Eagle’s medium (DMEM) with 20% fetal bovine serum (FBS). Human microvascular endothelial cells (Center for Disease Control) were expanded in 10% FBS in DMEM. All cell culture was performed with medium containing 1% penicillin/streptomycin at 37 °C and 5% CO_2_. Where indicated, mouse myoblasts were lentivirally transduced to express GFP under the control of ubiquitin promoter^46^. The schematic overview of cell seeding is shown in Supplementary Fig. 1. For in vitro studies, four conditions were assessed: (1) randomly oriented scaffold seeded with myoblasts; (2) randomly oriented scaffolds seeded with myoblasts + endothelial cells; (3) aligned scaffold seeded with myoblasts only; (4) aligned scaffold seeded with myoblasts + endothelial cells. Collagen scaffold strips (25 mm × 1 mm) were sterilized with 70% ethanol and rehydrated with three 20 min washes with 1× PBS. Mouse myoblasts (5 × 10^5^) were seeded into each scaffold and cultured in a high serum DMEM with 20% FBS. After 24 h (day 0), the growth medium was switched to DMEM containing 2% horse serum to induce differentiation and fusion of myoblasts. After 5 days of differentiation (day 5) during which myoblasts have fused to form myotubes, endothelial cells were co-cultured into the myoblast-seeded scaffolds (5 × 10^5^ cells per scaffold). For conditions with myoblasts only, no endothelial cells were added. For all conditions, media were switched to a maintenance medium (DMEM supplemented with 2% horse serum and 3% FBS), and constructs were culture for an additional 4 days (day 9) for in vitro testing (Supplementary Fig. 2).

### Immunofluorescence staining

Samples were fixed for 15 min in 4% paraformaldehyde (Alfa Aesar), washed three times with 1× PBS, and permeabilized in 0.5% Triton-X100 (Sigma) for 15 min. Samples were blocked in 1% bovine serum albumin (Sigma) and all subsequent steps were performed using 0.1% bovine serum albumin for antibody dilutions. For staining of actin, samples were incubated with Alexfluor-488-conjugated phalloidin (1:100, Life Technologies). For assessment of myotube morphology and maturity, fixed samples were incubated with a fast MHC marker (Abcam) for 16 h at 4 °C followed by Alexa Fluor-647 antibody (Life Technologies). For co-staining of endothelial cells, samples were then incubated with CD31 antibody (Dako) for 16 h at 4 °C followed by Alexa Fluor-594 antibody (Life Technologies). Images were acquired using a Zeiss LSM710 confocal microscope.

### Quantification of myotube morphology and maturity

Myoblasts were cultured on randomly oriented or aligned scaffolds with or without endothelial cells. To assess the impact of the scaffold patterning and the contribution of endothelial cells on myotube alignment, myotube length, nuclei per myotube, and percentage of striated myotubes (*n* = 3 each group), samples were fixed on day 9 and stained for MHC and CD31. Samples were acquired using a Zeiss LSM710 confocal microscope and z-stacked (100–200 μm stack) tile-stitched (5 × 2 frames) 20× images were taken. ImageJ (v1.46r) software and the angle tool were used to quantify cell orientation relative to a Cartesian axes with a range from 0° (cellular alignment parallel to nanofibrils) to 90° (cell alignment orthogonal to nanofibril orientation)^27^. Using the line and multipoint tools in ImageJ, myotube length and nuclei per myotube were measured, respectively. The percent of striated myotubes was assessed as number of MHC-expressing myotubes expressing visible striations out of total number of myotubes in which a myotube was defined as expressing GFP and containing ≥3 nuclei per myotube.

### Electrical stimulation to assess contractile function

Global contractile properties of engineered muscle formed from randomly oriented or aligned scaffolds, with or without endothelialization (*n* = 8 randomly oriented scaffold with no endothelialization, *n* = 6 aligned scaffold no endothelialization, *n* = 5 both groups with endothelialization) were evaluated by in vitro electrical pacing. On day 9 of in vitro culture, engineered muscle groups were stimulated at 1 Hz with 0.8 ms pulses of 50 V using platinum electrodes in a custom chamber kept at 37 °C. Scaffolds were positioned to capture the scaffold edge on the right-hand side of the frame window and were oriented horizontally such that the aligned nanofibers were in the horizontal direction. Bright field images were taken at 25 frames per second to collect 10-s movies using a custom Labview script and Axio Vision software (Supplementary Movie S1, S2). Image analysis was performed using the ImageJ Particle Image Velocimetry plugin for magnitude-vector plots and a custom MATLAB script for tracking particle movement to measure percent area contraction and max contraction velocity^57^.

### Cytokine proteome arrays

Cell culture supernatants (1 ml each sample) were collected engineered skeletal muscle formed from randomly oriented or aligned scaffolds, with or without endothelialization, on days 6 and 9 of in vitro culture (*n* = 3 aligned scaffold day 9, *n* = 4 all other groups). A human-specific angiogenic cytokine proteome profiler array (R&D Systems) was utilized according to manufacturer’s instructions, to assess the relative expression of angiogenic cytokines by endothelial cells. Radiographic film (Kodak) was exposed to chemiluminescent samples for 10 min in the dark, followed by development with an automatic film processor machine. Films were scanned at 600 dots per inch with a flatbed transmission scanner and converted to gray scale in ImageJ software. Image pixels were inverted such that white pixels were given a value of 255 and black pixels a value of 0. Using the circle tool, integrated density (mean pixel value per area) was measured for each spot on the array and the identity of each cytokine was matched using the manufacturer’s provided array legend. Duplicate spots for each cytokine were averaged, and the mean values of all experimental samples (*n* = 3 aligned scaffold day 9, *n* = 4 all other groups) were determined for each cytokine and condition. The data are expressed as relative fold change compared with engineered skeletal muscle in randomly oriented scaffolds on day 6.

### VEGF inhibition studies

To assess the role of spatially patterned scaffolds in the secretion of VEGF, endothelial cells were seeded on either aligned or randomly oriented scaffolds (*n* = 3) containing differentiated myotubes and cultured together for 4 days in maintenance medium (DMEM supplemented with 2% horse serum and 3% FBS), after which they were incubated for 24 h with a VEGF receptor tyrosine kinase inhibitors, AAL-993 (EMD Millipore, 1 µM) or axitinib (Selleck Chemicals, 10 µM)^58^ in maintenance medium. After incubation with the inhibitor, the cells were assessed for angiogenic function based on the production of NO using the fluorescent probe, 4-amino-5-methylamino-2′,7′-difluorofluorescein diacetate (DAF-FM, Fisher Scientific a cell-permeable, fluorescent probe for the detection and imaging of NO) production. Samples were incubated with DAF-FM (5 μm in maintenance medium) for 30 min followed by incubation in maintenance medium for 20 min. Media were replaced with fresh maintenance media and samples were imaged immediately by confocal microscopy (Zeiss LSM710). For each sample, three representative fluorescent images were taken at 10×. Integrated density of DAF-FM expression by the endothelial cells (mRuby^+^ area) was calculated using a set cut-off threshold of 60 in ImageJ (Supplementary Fig. 3).

### RNA sequencing

RNA was isolated from cell lysates from engineered muscle and endothelialized engineering muscle on randomly oriented (*n* = 3) or aligned scaffolds (*n* = 3 and *n* = 2, respectively) according to the manufacturer’s protocol (GeneJet RNA Purification Kit, Thermo Fisher). At least 1000 ng of total RNA was pelleted per sample. Library preparation and RNA Sequencing was performed by Novogene Corporation using the NovaSeq 6000 (Illumina) sequencing platform, and 150-bp paired-end reads were generated (20–30 million reads per sample). Briefly, RNA integrity was assessed (RNA Nano 6000 Assay Kit, Bioanalyzer 2100, Aligent Technologies, CA) and 3 µg RNA per sample was used to generate sequencing libraries (NEBNext Ultra RNA Library Prep Kit for Illumina) according to manufacturer’s recommendations. Library fragments were purified (AMPure XP system, Beckman Coulter, Beverly) to select complementary DNA (cDNA) fragments (150–200 bp in length). PCR was performed with Phusion High-Fidelity DNA polymerase, and purified by the AMPure XP system and assessed for quality on the Agilent Bioanalyzer 2100 system. The raw data were checked for quality with FastQC (Version 0.11.7) and results were aggregated with MultiQC and were aligned to the mouse genome (GRCm38) using STAR (Version 2.5.3a) with ENCODE options for long RNA-Seq pipeline. The alignment results were assessed using Samtools and aggregated with MultiQC (Version 1.5) and the differential gene expression analysis of the uniquely mapped reads/raw counts was performed using the DESeq2 package (Version 1.20.0). Each differential expression analysis was composed of a pairwise comparison between an experimental group and the control group. Differentially expressed genes were identified after false discovery rate (FDR = 0.05) correction.

### GSEA

The mouse symbols were first converted to corresponding human homologs/orthologs. Mouse symbols without corresponding human homologs/orthologs were not included for GSEA. Gene ranks for pre-ranked GSEA were defined as -log10(padj) for upregulated genes (log2(fold change) >0) and (−1) × −log10(padj) for downregulated genes, where padj is the Benjamini–Hochberg corrected *p*-value from DESeq2. The changes of these genes were used to generate a ranked list for GSEAPreranked analysis using the Molecular Signatures Database v5.2 (H, hallmark gene sets). For hallmark analysis, the top 10 significantly enriched gene sets ranked by GSEA normalized enrichment score were visualized. Gene sets with an FDR < 0.05 were defined as significantly enriched.

### Implantation of engineered muscle into injured mouse muscle

For transplantation in vivo, 3D engineered murine skeletal muscle bundles were fabricated. In brief, endothelialized engineered skeletal muscle strips were formed as described above. On day 9 of in vitro culture, eight scaffold strips were gently detached from the slide supports and allowed to self-organize into a 3D bundle that was cut to approximately 9 mm × 2 mm × 3 mm (Supplementary Fig. 6).

Volumetric muscle loss was induced in NOD SCID mice (male, 8 weeks old, Jackson Laboratories) by surgical excision of 20% of the tibialis anterior muscle^34^. Immediately afterward, one of the following treatment groups was implanted at the site of the muscle defect: (1) engineered muscle in randomly oriented scaffold; (2) endothelialized engineered muscle in randomly oriented scaffold; (3) engineered muscle in aligned scaffold; (4) endothelialized engineered muscle in aligned scaffold; or (5) aligned scaffold without cells (*n* = 7 per group). In these studies, the murine myoblasts within the engineered skeletal muscle were tagged with dual reporters GFP and firefly luciferase for invasive tracking using fluorescence and bioluminescence imaging, respectively. In a separate set of studies, only the endothelial cells within the engineered skeletal muscle were tagged with dual reporters mCherry and firefly luciferase for in vivo transplantation (*n* = 4). All animal studies were approved by the Institutional Animal Care and Use Committee at the Veterans Affairs Palo Alto Health Care System.

### Bioluminescence imaging of cell survival

Cell survival and localization was monitored by bioluminescence imaging (*n* = 4 each group) on days 0, 7, 14, and 21 by intraperitoneal injection of d-luciferin, followed by imaging in an IVIS Spectrum (Xenogen). The data were expressed in units of average radiance (p s^−1^ cm^−2^ sr^−1^). As the release of photons require that the cells are viable, this non-invasive imaging approach measures the photons released only by viable transplanted cells.

### Histological analysis of blood perfusion

After 21 days following implantation, animals were injected via the tail vein with 100 µl (1 mg ml^−1^) of endothelial-binding fluorescent isolectin GS-IB4 (Invitrogen) 25 min prior to euthanasia, followed by explantation of the tibialis anterior muscle. The tibialis anterior muscles were fixed in 0.4% paraformaldehyde at 4 °C under continuous gentle agitation for 16 h. After fixation, samples underwent density equilibration in 20% sucrose for 2–3 h and followed by embedding for cryosectioning of tissue sections in the transverse or longitudinal planes. Histological quantification of blood perfusion was performed by immunofluorescence staining with endothelial marker, CD31 (R&D Systems). Five non-overlapping images (500 µm × 500 µm) from transverse cryosections for each animal (*n* = 4 each group except *n* = 6 endothelialized engineered muscle with randomly oriented scaffold) were taken within 500 µm from the boarders of the transplanted scaffolds. Vessels that were CD31^+^ and co-stain with isolectin indicated vessels that were functionally anastomosed to the host circulation. The perfused vessel density was expressed as the total number of perfused vessels mm^−2^. Vessel regeneration was also quantified similarly for acellular transplanted scaffolds (*n* = 4). To assess the orientation of vascular networks, longitudinal tissue sections adjacent to the scaffold were cryosectioned at a 50 µm thickness (*n* = 3 each group) and then immunofluorescently stained with MHC. Tiled *z*-stacked images (3 × 7 montage using 20× objectives) were taken using confocal microscopy to visualize the organization of donor-derived myofibers based on the dual expression of GFP and MHC, as well as the organization of isolectin^+^ vessels, along the length of the tibialis anterior muscle.

The organization of the vasculature was assessed using the FibrilTool, an ImageJ macro that calculates the overall degree of spatial patterning among the vessels. Using three confocal images per sample stained with CD31 antibody (*n* = 4 each group) the vessel organization was quantified, whereby a score of 0 denotes the absence of spatial patterning, and 1 denotes parallel alignment of the vessels. To further substantiate the spatial organization of the vessels, two-dimensional Fast Fourier Transform analysis was performed using an ImageJ plugin to generate frequency plots^59,60^. In addition, longitudinal sections from the interface between the host and transplanted tissue were imaged by confocal microscopy for MHC, GFP, and Hoechst 33342 nuclear dye. Quantification of anisotropy and the global angle of alignment was performed using the FibrilTool plugin for ImageJ and a single region of interest was taken as the entire 10× image.

### Immunofluorescence analysis of muscle regeneration

Histological analysis of tissue morphology was performed on serial transverse cryosections (10 µm thickness) of the tibialis anterior muscle. Samples were stained with hematoxylin and eosin or Trichrome. To quantify myofiber regeneration within the scaffold’s immediate vicinity (within 500 µm from the scaffold’s borders), muscle tissue cryosections were immunofluorescently stained with MHC, and tiled *z*-stacked images (5 × 5 montages using 20× objectives) were taken using confocal microscopy to capture the entire cross-section of the tissue. Using the multipoint tool in ImageJ, the total number of donor-derived myofibers that dually expressed GFP and MHC myofibers in the vicinity of the scaffold implants was counted (*n* = 3 engineered muscle, randomly oriented scaffold; *n* = 5 engineered muscle, aligned scaffold; *n* = 7 endothelialized muscle both groups).

### Engineered skeletal muscle composed of primary human cells

The primary human muscle precursor cells were cultured in skeletal growth medium-2 (SkGM2), and primary human microvascular endothelial cells were cultured in endothelial growth medium-2 (EGM2-MV) (all from Lonza). For in vitro studies, four conditions were assessed: (1) randomly oriented scaffold seeded with human muscle precursor cells; (2) randomly oriented scaffold seeded with human muscle precursor cells + primary human microvascular endothelial cells; (3) aligned scaffold seeded with human muscle precursor cells; (4) aligned scaffold seeded with human muscle precursor cells + primary human microvascular endothelial cells. For cell seeding studies, collagen scaffolds were sterilized with 70% ethanol and rehydrated with three washes with 1× PBS for 20 min.

The primary human muscle precursor cells were seeded (1 × 10^6^ per scaffold) and cultured in SkGM2 media. After 24 h (day 0), the medium was switched to 2% reduced serum differentiation medium (DMEM + 2% horse serum) to induce fusion of myoblasts. After 5 days of differentiation (day 5) during which human muscle precursor cells had fused to form myotubes, the primary human microvascular endothelial cells (1 × 10^6^ cells per scaffold) were added to the scaffolds. For conditions with primary human muscle precursor cells only, no primary human microvascular endothelial cells were added. For all conditions, media were switched to a maintenance medium (25% relative ratio of DMEM containing 2% horse serum, 75% EGM2-MV) and engineered muscle were cultured for an additional 4 days until day 9. Engineered muscle groups were then fixed in 4% paraformaldehyde for immunostaining to assess morphology and cellular organization as described above.

### Immunofluorescence staining and quantitative PCR analysis

Cell lysates were collected from human muscle precursor cells cultured for 48 h in expansion medium and RNA was isolated using a GeneJET RNA Purification kit (Thermo Fisher) according to manufacturer’s instructions with modifications. Total RNA from adult human skeletal muscle was purchased from Fisher Scientific (AM7982). The cDNA was synthesized using Superscript II reverse transcriptase (Life Technologies). Quantitative real-time PCR was performed on a 7300 Real-Time PCR system (Life Technologies). Taqman primers consisted of Pax7 (Hs00242962_m1) and housekeeping gene GAPDH (HS99999905_m1). Relative fold change in gene expression was calculated using the ΔΔCt method and normalized to GAPDH housekeeping gene expression.

To confirm expression of Pax7, undifferentiated human muscle precursor cells were fixed with 4% parformaldehyde followed by immunofluorescent staining as described above. Samples were incubated with Pax7 (DSHB) for 16 h at 4 °C followed by Alexa Fluor-594 antibody (Life Technologies). For co-staining of F-actin, samples were then incubated with Alexa Fluor Phalloidin-488 (Dako) for 16 h at 4 °C.

The human muscle precursor cells were cultured on randomly oriented or aligned scaffolds, forming engineered skeletal muscle with or without endothelialization by primary human microvascular endothelial cells. To assess the impact of the scaffold patterning and the contribution of primary human microvascular endothelial cells on myotube alignment, engineered muscle samples (*n* = 3 each group) were fixed on day 9 and co-stained using MHC and CD31 antibodies. Samples were acquired using a Zeiss LSM710 confocal microscope and *z*-stacked (100–200 μm stack) tile-stitched (5 × 2 frames) 20× images were taken. ImageJ software and the angle tool were used to quantify cell orientation relative to a Cartesian axes with a range from 0° (cellular alignment parallel to nanofibrils) to 90° (cell alignment orthogonal to nanofibril orientation)^27^.

### Transplantation of primary human engineered skeletal muscle

For transplantation in vivo, a 3D engineered human skeletal muscle bundle was fabricated. In brief, endothelialized engineered skeletal muscle strips were formed as described above. On day 9, eight scaffold strips were gently detached from the slide supports and allowed to self-organize into a 3D bundle that was cut to approximately 9 mm × 2 mm × 3 mm. The human engineered skeletal muscle bundles were transplanted into the ablated tibialis anterior muscle of SCID mice, as described above, for assessment of cell survival, muscle regeneration, and revascularization for 21 days at the site of the muscle defect: (1) endothelialized engineered muscle formed from randomly oriented scaffold; or (2) endothelialized engineered muscle formed from aligned scaffold (*n* = 6 each group).

### Tissue regeneration and revascularization

To quantify myofiber regeneration within the scaffold’s vicinity, muscle tissue cryosections were immunofluorescently stained with human nuclear antigen (Millipore) and tiled *z*-stacked images (5 × 5 montage using 20× objectives) were taken using confocal microscopy to capture the entire cross-section of the tissue. Human donor-derived myofibers were visualized by the dual expression of human nuclear antigen and MHC. Using the multipoint tool in ImageJ, the total number of human donor-derived myofibers in the region of regenerating myofibers within 500 µm from the scaffold borders was counted (*n* = 6). Vascular perfusion was quantified as described above by co-staining of tibialis anterior muscle tissues for CD31^+^/isolectin^+^ dual expression (*n* = 3). Perfused vessel density data were shown as the total number of perfused vessels mm^−2^.

### Statistical analysis

All statistical analysis was performed using GraphPad PRISM software. Where appropriate, a one-way analysis of variance (ANOVA) or two-way ANOVA was performed with post hoc Tukey’s adjustment. For comparison between two groups only, an unpaired two-tailed Student’s *t*-test was used. Significance was taken at *p* ≤ 0.05 (*), *p* ≤ 0.01 (**), *p* ≤ 0.001 (***), and *p* ≤ 0.0001 (****). All graphs were made in either Microsoft Excel or GraphPad PRISM and display mean ± standard deviation (SD).

### Reporting summary

Further information on research design is available in the Nature Research Reporting Summary linked to this article.

## Supplementary information

Supplementary Figures

Description of Additional Supplementary Files

Supplementary Data 1

Reporting Summary

Supplementary Movie 1

Supplementary Movie 2

## Data Availability

RNA sequencing data have been deposited to the Gene Expression Omnibus (GSE127171). The source data underlying the main figures are available as Supplementary Data 1. All other data supporting the conclusions of this paper are available from the corresponding author upon reasonable request.

## Code availability

The custom MATLAB code for measurement of in vitro contractile function can be found at the Stanford Digital Repository (https://purl.stanford.edu/jm601xh1638)^57^.
