# Supplementary material for: Treatment of volumetric muscle loss in mice using nanofibrillar scaffolds enhances vascular organization and integration
Source: Commun Biol. 2019 May 7;2:170. doi: 10.1038/s42003-019-0416-4 (PMC6505043; doi:10.1038/s42003-019-0416-4)
Supplement: Supplementary file 1 — Supplementary Figures [file 42003_2019_416_MOESM1_ESM.pdf]

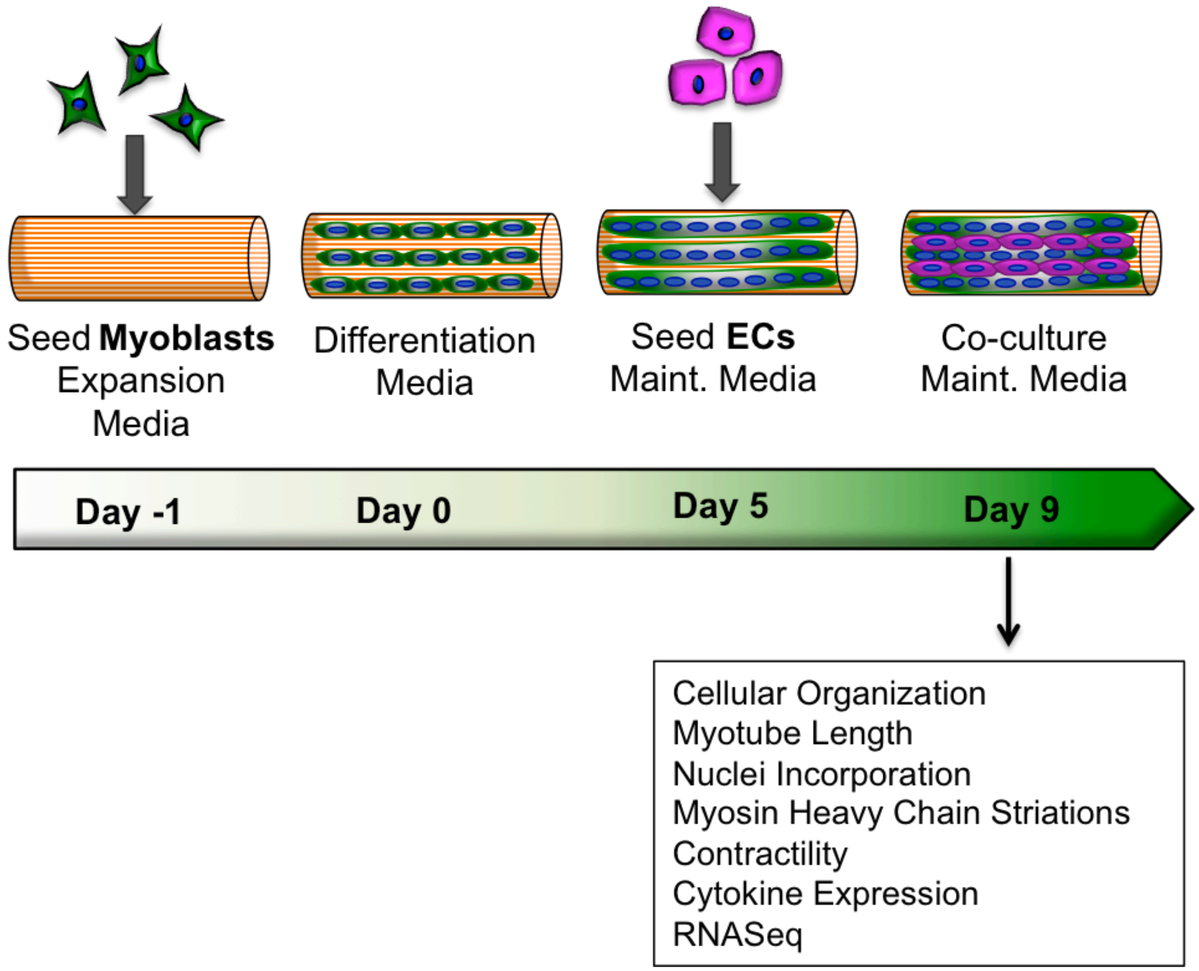

Supplementary Figure 1. Overview schematic of the *in vitro* experiments. Endothelialized engineered skeletal muscle constructs were used for *in vitro* assessment of cellular organization, contractility, transcriptional profiling, and cytokine expression profiling.

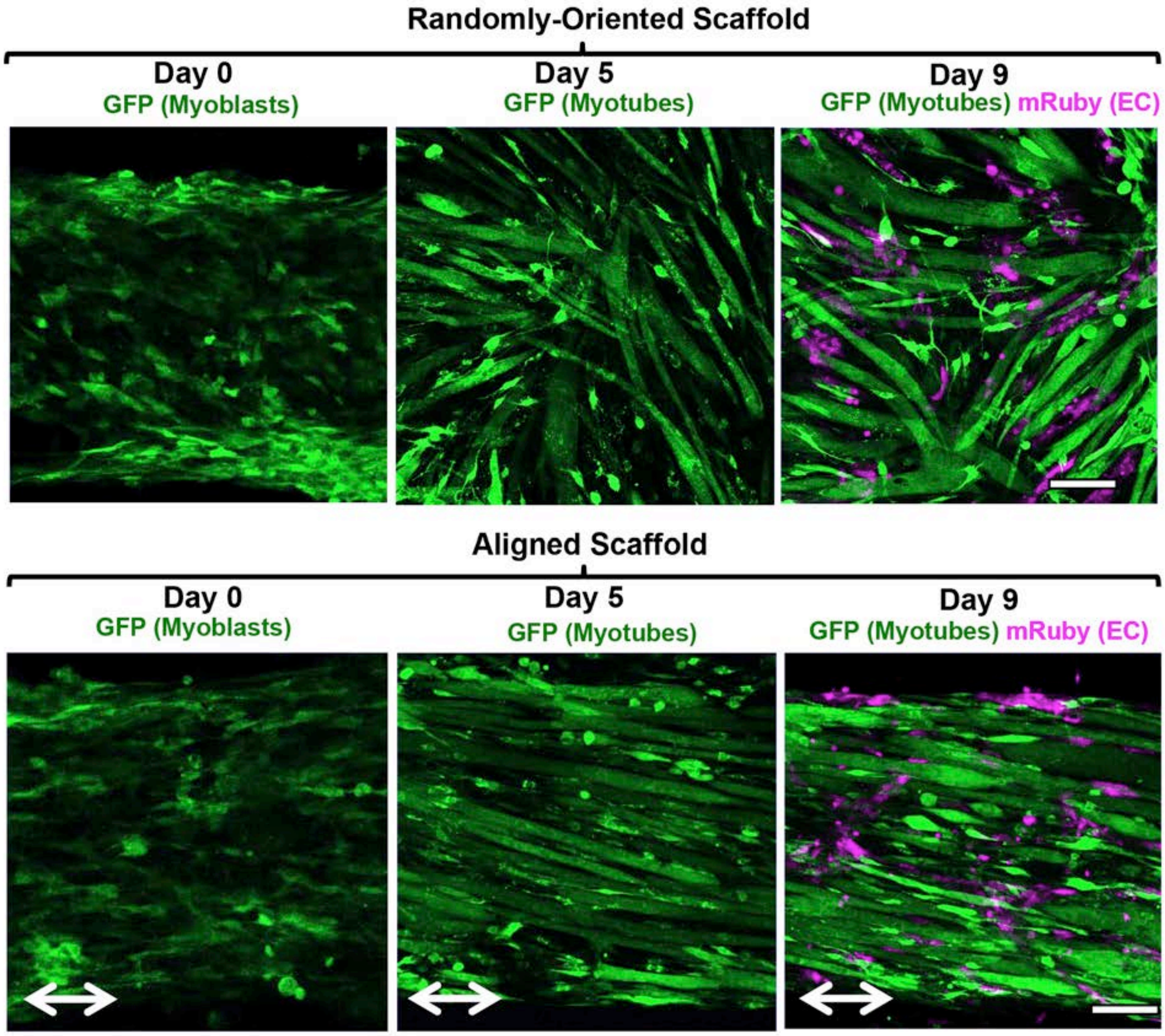

Supplementary Figure 2. Generation of endothelialized engineered skeletal muscle. Differentiation of green fluorescence protein-expressing mouse myoblasts (green, day 0) into fused myotubes (day 5), followed by co-culture with mRuby-expressing endothelial cells (magenta) until day 9. White arrows denote orientation of scaffold fibers. Scale bar denotes 100 µm.

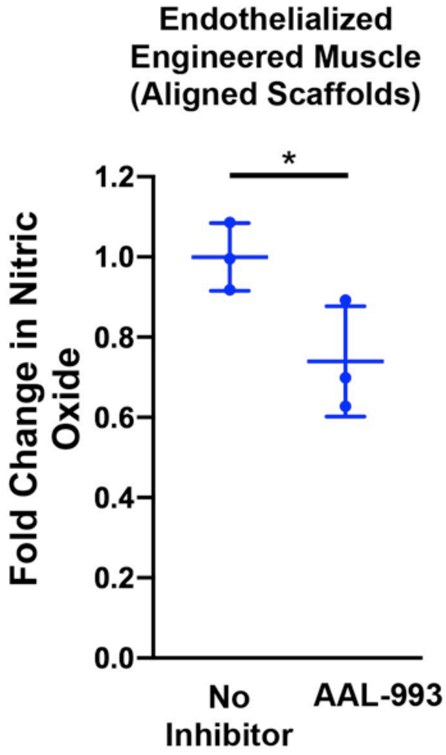

Supplementary Figure 3. Effect of vascular endothelial growth factor receptor (VEGFR) inhibition on nitric oxide production within endothelialized engineered muscle derived from aligned or randomly oriented scaffolds. The effect of VEGFR inhibitor (AAL-993, 1 μM) on nitric oxide production in endothelial cells cultured on endothelialized engineered muscle derived from aligned or randomly oriented scaffolds. The level of nitric oxide is expressed as a fold change relative to no treatment control, as denoted by the dotted line (*p≤0.05, n=3).

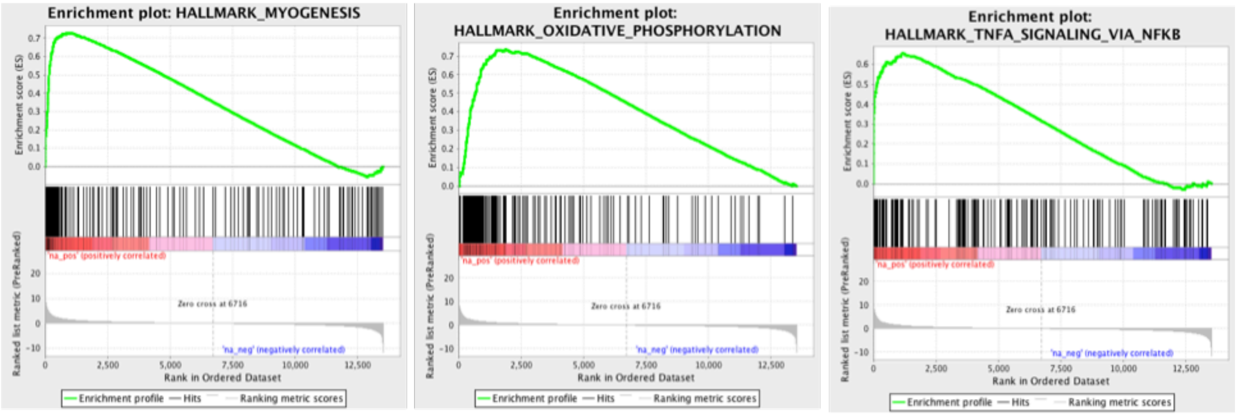

Supplementary Figure 4. Transcriptional analysis of non-endothelialized engineered muscle derived from randomly oriented or aligned scaffolds. Gene Set Enrichment Analysis showing selected Gene Ontology data sets enriched in the non-endothelialized engineered muscle grown on aligned scaffolds, in comparison to randomly oriented scaffolds (n=3).

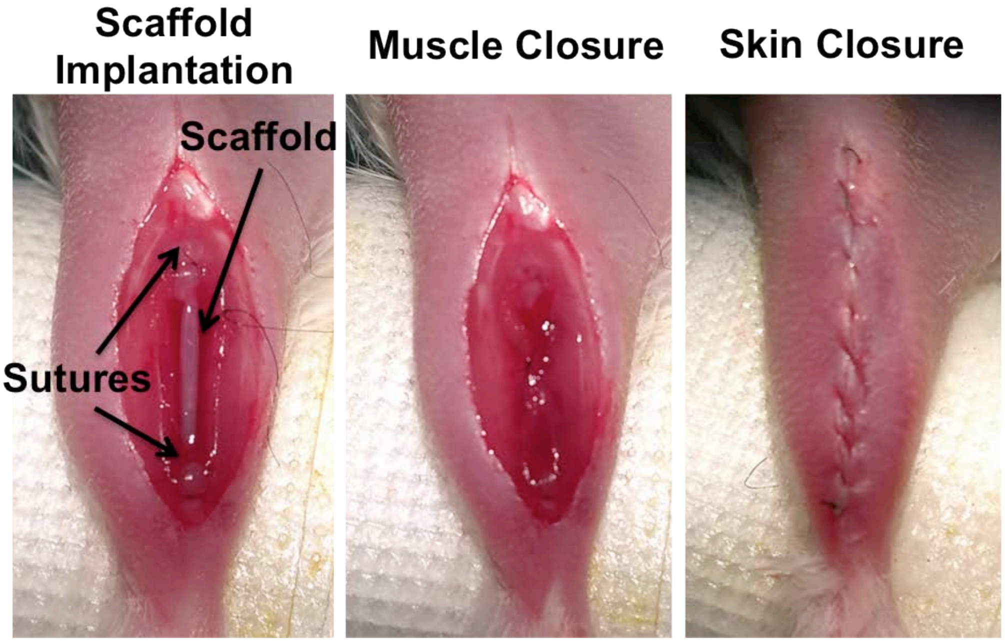

Supplementary Figure 5. Intraoperative images of muscle ablation, followed by transplantation of engineered skeletal muscle. Shown are images depicting surgical induction of muscle injury by partial ablation of the tibialis anterior muscle, followed by transplantation of engineered skeletal muscle. Afterwards, the muscle was sutured closed, followed by closure of the skin.

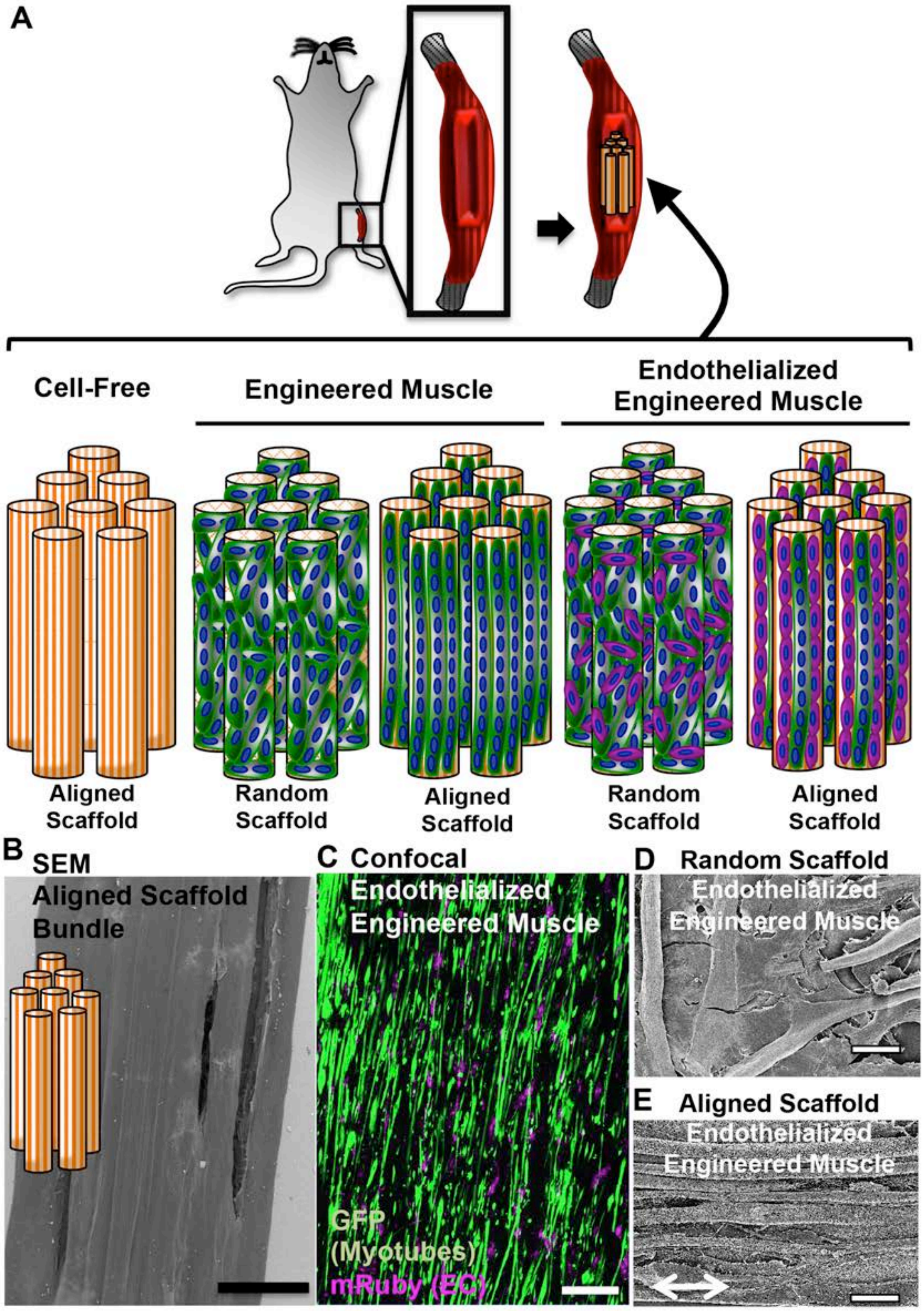

Supplementary Figure 6. Schematic overview and characterization of engineered skeletal muscle. (**A**) Schematic diagram of the engineered skeletal muscle groups for transplantation into

the ablated tibialis anterior muscle. (**B**) Scanning electron microscopy (SEM) images of acellular aligned scaffold bundle on day 9 of *in vitro* culture. Inset is an illustration of the bundle organization. Scale bar denotes 500 μm. (**C**) Endogenous expression of green fluorescence protein (GFP, green) by myotubes and mRuby (magenta) by endothelial cells, respectively. Scale bar denotes 500 μm. SEM images of endothelialized engineered skeletal muscle formed from (**D**) randomly-oriented and (**E**) aligned scaffolds. White arrows denote the orientation of scaffold nanofibers. Scale bar denotes 20 μm.

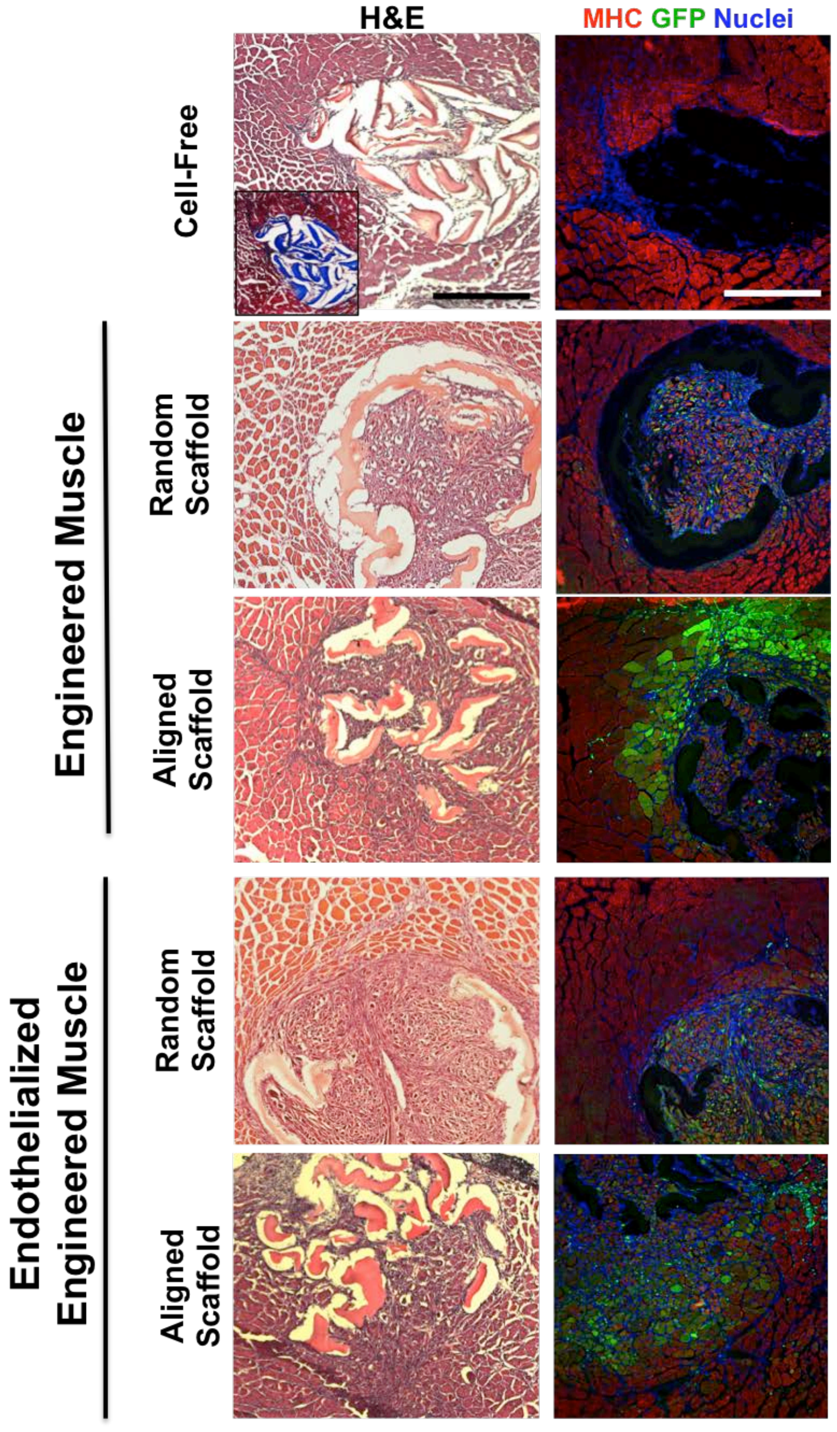

Supplementary Figure 7. Histology of transplanted engineered skeletal muscle. Left Column depicts hematoxylin and eosin (H&E) staining in transverse tissue sections of the muscle

adjacent to the transplant site at 21 days after implantation into mice with volumetric muscle loss. The inset shows trichrome stain depicting the partially degraded collagen scaffold in blue color. The right column depicts immunofluorescence staining of myosin heavy chain (MHC, red) and green fluorescence protein (GFP, green) surrounding the transplant site. Scale bar denotes 500 μm.

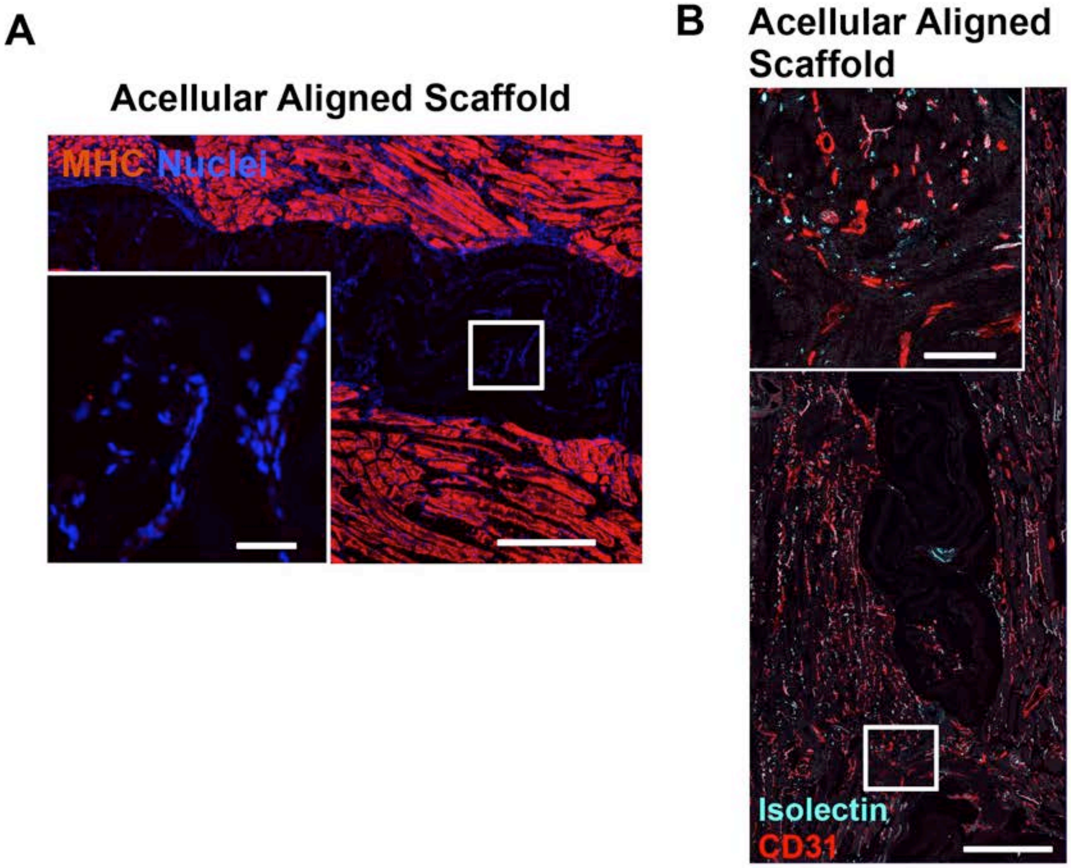

Supplementary Figure 8. Transplantation of acellular scaffold into the ablated mouse muscle after 21 days. (**A**) Representative longitudinal tissue sections adjacent to the site of the implanted acellular aligned scaffold. Myosin heavy chain (MHC, red) for mature myofibers and Hoechst 33342 dye (blue) for total nuclei are shown ($n \geq 3$). The inset shows a high magnification image of the region depicted by white box. Scale bar (Inset) denotes 50 µm, Scale bar denotes 500 µm. (**B**) Immunofluorescent staining of CD31 (red) and isolectin (turquois) in transverse tissue sections adjacent to the implanted acellular scaffold. Inset shows a high magnification image of the region depicted by white box. Scale bar (Inset) denotes 100 µm, Scale bar denotes 500 µm.

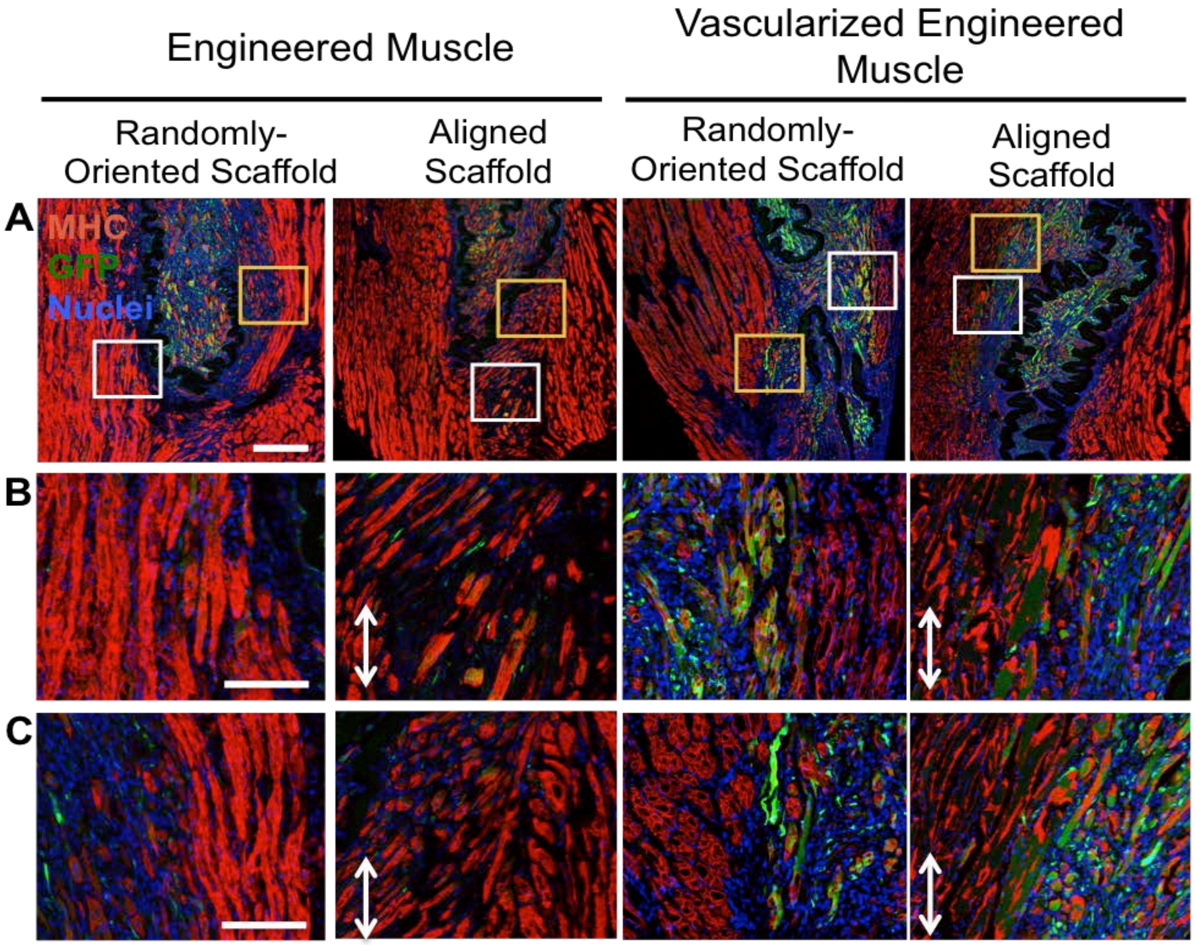

Supplementary Figure 9. Murine engineered skeletal muscle integration into a mouse model of volumetric muscle loss. (**A**) Representative longitudinal sections of endothelialized engineered muscle formed randomly-oriented or aligned skeletal muscle constructs at 21 days after induction of traumatic muscle injury. Myosin heavy chain (MHC, red) indicates mature myofibers, green fluorescence protein (GFP, green) indicates transplanted myoblast population, and nuclei are visualized using Hoechst 33342 dye (blue). The organization of transplanted myofibers (MHC^+/GFP^+) at the distal ends of the engineered muscle is shown ($n \geq 3$). Scale bar denotes 500 µm. (**B, C**) Higher magnification images of regions depicted by the white and yellow boxes, respectively, in (**A**) are shown. Arrows denote orientation of aligned nanofibrillar scaffolds. Scale bar denotes 200 µm.

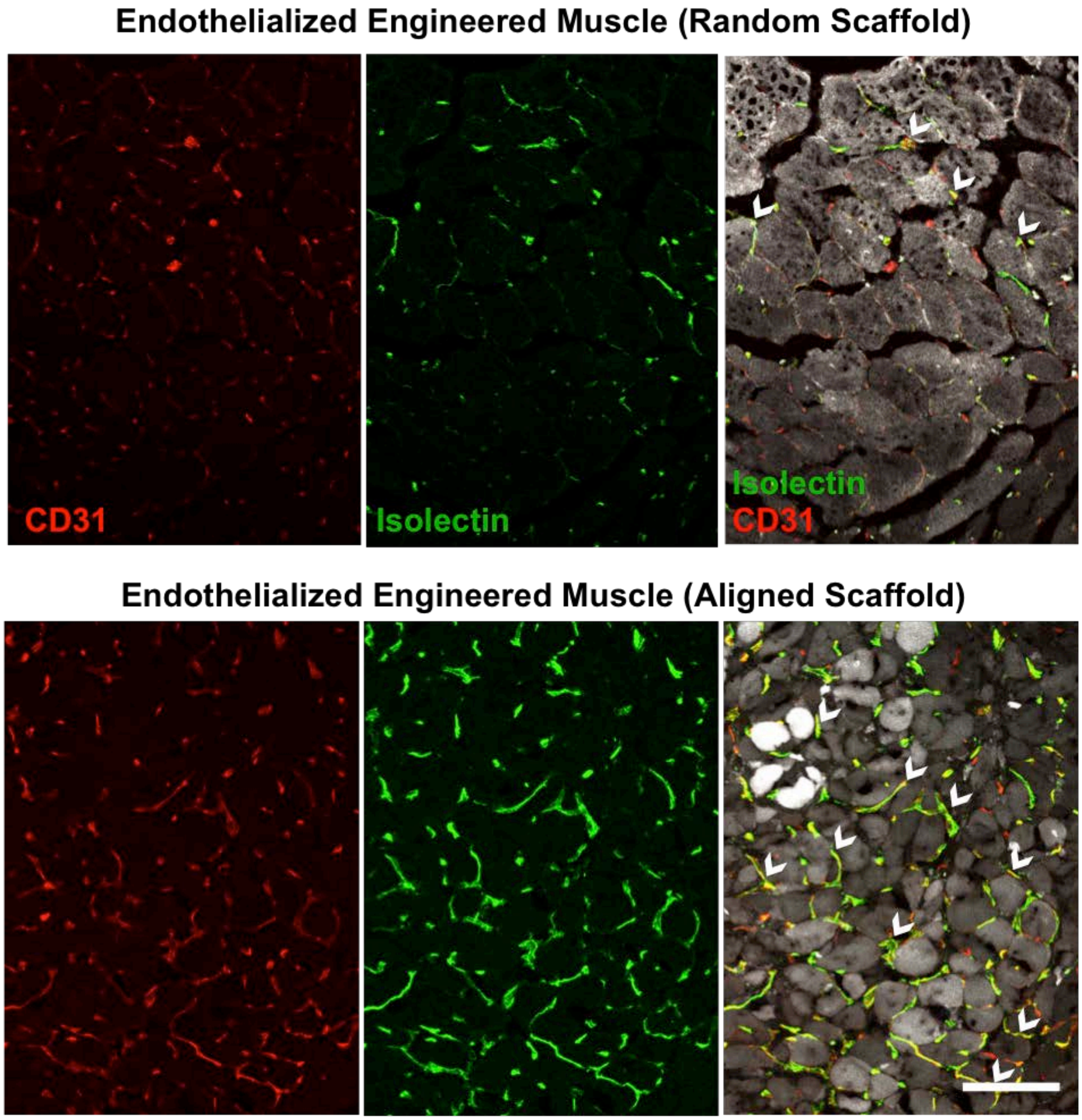

Supplementary Figure 10. Vascular perfusion of transplanted endothelialized skeletal muscle. Immunofluorescent staining of CD31 (red) and isolectin (green) in transverse tissue sections adjacent to the implanted engineered muscle at 21 days after implantation into traumatically injured muscle. Arrowheads denote examples of CD31$^+$/isolectin$^+$ vessels. Scale bar denotes 100 μm.

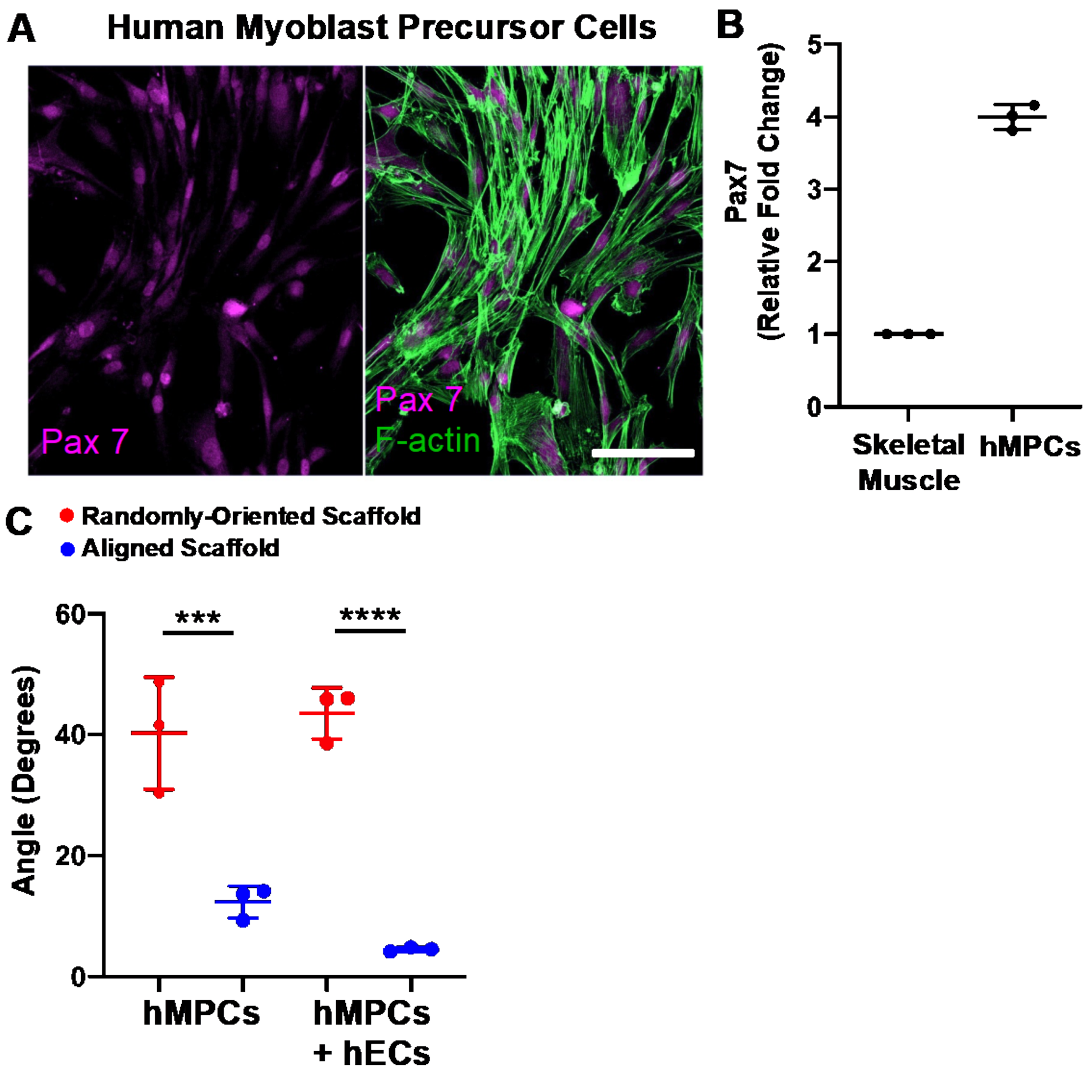

Supplementary Figure 11. Characterization of engineered muscle derived from primary human cells. (**A**) Immunofluorescence staining of Pax7 (magenta) and F-actin (green) in primary human muscle progenitor cells (hMPCs) grown in tissue culture dish. Scale bar denotes 100 µm. (**B**) Normalized gene expression of Pax7 in hMPCs, expressed relative to primary human skeletal muscle tissue. (**C**) Engineered human muscle was formed by seeding of hMPCs and primary human endothelial cells (hECs) in either randomly oriented or aligned scaffolds. The angle of orientation of differentiated myotubes after 9 days is depicted (n=3, ***p<0.001, ****p<0.0001).

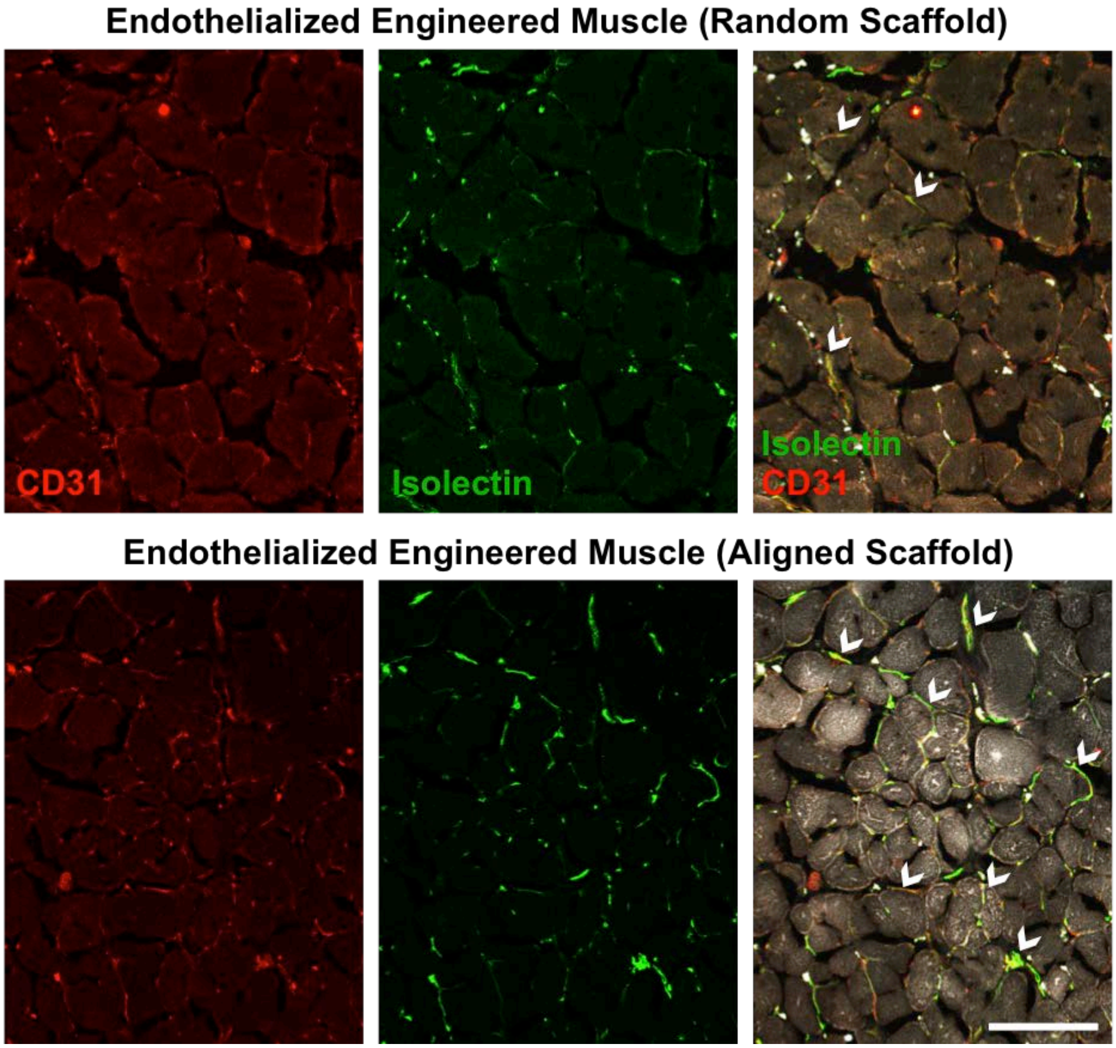

Supplementary Figure 12. Vascular perfusion of transplanted human endothelialized engineered skeletal muscle. Immunofluorescence staining of CD31 (red) and isolectin (green) adjacent to the transplantation site. Scale bar denotes 100 μm.
