# Supplementary material for: Treatment of volumetric muscle loss in mice using nanofibrillar scaffolds enhances vascular organization and integration
Source: Commun Biol. 2019 May 7;2:170. doi: 10.1038/s42003-019-0416-4 (PMC6505043; doi:10.1038/s42003-019-0416-4)
Supplement: Supplementary file 2 — Description of Additional Supplementary Files [file 42003_2019_416_MOESM2_ESM.pdf]

Description of Additional Supplementary Files

File Name: Supplementary Movie 1

Description: Electrically stimulated endothelialized skeletal muscle formed from randomly oriented scaffolds. Representative movie of engineered skeletal muscle after 9 days of culture during electrical stimulation (1 Hz). The movie captures the edge of the engineered muscle (far right).

File Name: Supplementary Movie 2

Description: Electrically stimulated endothelialized skeletal muscle formed from aligned scaffolds. Representative movie of engineered skeletal muscle after 9 days of culture during electrical stimulation (1 Hz). The movie captures the edge of the engineered muscle (far right). The nanofibrils orientation is parallel to the horizontal axis.

File Name: Supplementary Data 1

Description: The Supplementary Data 1 file contains all source data underlying the graphs and charts presented in the main figures (Figure 1-9). The source data includes: Myotube formation and orientation; Engineered muscle contractility; Cytokine production, Nitric oxide production; Bioluminescence intensity; RNA Sequencing data; Histological assessment of donor-derived myofibers density and vascular density; and microvascular orientation.
