# Supplementary material for: Treatment of volumetric muscle loss in mice using nanofibrillar scaffolds enhances vascular organization and integration
Source: Commun Biol. 2019 May 7;2:170. doi: 10.1038/s42003-019-0416-4 (PMC6505043; doi:10.1038/s42003-019-0416-4)
Supplement: Supplementary file 4 — Reporting Summary [file 42003_2019_416_MOESM4_ESM.pdf]

nature research

Corresponding author(s): Ngan Huang, COMMSBIO-17-0201A

Last updated by author(s): Mar 18, 2019

Reporting Summary

Nature Research wishes to improve the reproducibility of the work that we publish. This form provides structure for consistency and transparency in reporting. For further information on Nature Research policies, see Authors & Referees and the Editorial Policy Checklist.

Statistics

For all statistical analyses, confirm that the following items are present in the figure legend, table legend, main text, or Methods section.

n/a	Confirmed	
☐	☒	The exact sample size (*n*) for each experimental group/condition, given as a discrete number and unit of measurement
☐	☒	A statement on whether measurements were taken from distinct samples or whether the same sample was measured repeatedly
☐	☒	The statistical test(s) used AND whether they are one- or two-sided<br>*Only common tests should be described solely by name; describe more complex techniques in the Methods section.*
☐	☒	A description of all covariates tested
☐	☒	A description of any assumptions or corrections, such as tests of normality and adjustment for multiple comparisons
☐	☒	A full description of the statistical parameters including central tendency (e.g. means) or other basic estimates (e.g. regression coefficient) AND variation (e.g. standard deviation) or associated estimates of uncertainty (e.g. confidence intervals)
☒	☐	For null hypothesis testing, the test statistic (e.g. *F*, *t*, *r*) with confidence intervals, effect sizes, degrees of freedom and *P* value noted<br>*Give P values as exact values whenever suitable.*
☒	☐	For Bayesian analysis, information on the choice of priors and Markov chain Monte Carlo settings
☒	☐	For hierarchical and complex designs, identification of the appropriate level for tests and full reporting of outcomes
☒	☐	Estimates of effect sizes (e.g. Cohen's *d*, Pearson's *r*), indicating how they were calculated

Our web collection on statistics for biologists contains articles on many of the points above.

Software and code

Policy information about availability of computer code

Data collection	All graphs were made in either Microsoft Excel or GraphPad PRISM. All statistical analysis was performed using Graph Pad PRISM software.
Data analysis	All quantitative analysis of raw data was performed using ImageJ, Labview and MATLAB. A reference to the custom MATLAB code has been provided. The raw data were checked for quality with FastQC (Version 0.11.7) and results were aggregated with MultiQC and were aligned to the mouse genome (GRCm38) using STAR (Version 2.5.3a) with ENCODE options for long RNA-Seq pipeline. The alignment results were assessed using Samtools and aggregated with MultiQC (Version 1.5) and the differential gene expression analysis of the uniquely mapped reads/raw counts was performed using the DESeq2 package (Version 1.20.0).

For manuscripts utilizing custom algorithms or software that are central to the research but not yet described in published literature, software must be made available to editors/reviewers. We strongly encourage code deposition in a community repository (e.g. GitHub). See the Nature Research guidelines for submitting code & software for further information.

Data

Policy information about availability of data

All manuscripts must include a data availability statement. This statement should provide the following information, where applicable:

- Accession codes, unique identifiers, or web links for publicly available datasets
- A list of figures that have associated raw data
- A description of any restrictions on data availability

The datasets generated during and/or analyzed during the current study are available from the corresponding author on reasonable request. RNA Sequencing data have been deposited to the Gene Expression Omnibus (GSE127171). The source data underlying the main figures are available as Supplementary Data. All other data supporting the conclusions of this paper are available from the corresponding author upon reasonable request.

Field-specific reporting

Please select the one below that is the best fit for your research. If you are not sure, read the appropriate sections before making your selection.

☒ Life sciences ☐ Behavioural & social sciences ☐ Ecological, evolutionary & environmental sciences

For a reference copy of the document with all sections, see nature.com/documents/nr-reporting-summary-flat.pdf

Life sciences study design

All studies must disclose on these points even when the disclosure is negative.

Sample size	The samples sizes were determined by power calculations to achieve power of 0.8, based on expected differences observed from preliminary observations and studies.
Data exclusions	Exclusion of data was done in the case in which histological samples were determined to be unusable due to poor tissue quality due to damage to the tissue structure.
Replication	All findings were replicated in at least 3 independent experiments and results were found to be consistent between experiments.
Randomization	Samples were randomized between groups. Sample consistency was verified by multiple SEM sessions and phase contrast microscopy prior to inclusion. Samples with no visible differences were included and randomized at the start of the study.
Blinding	Investigators were blinded to the identification of groups during analysis based on sample naming methodology

Reporting for specific materials, systems and methods

We require information from authors about some types of materials, experimental systems and methods used in many studies. Here, indicate whether each material, system or method listed is relevant to your study. If you are not sure if a list item applies to your research, read the appropriate section before selecting a response.

Materials & experimental systems

n/a	Involved in the study	
☐	☒	Antibodies
☐	☒	Eukaryotic cell lines
☒	☐	Palaeontology
☐	☒	Animals and other organisms
☒	☐	Human research participants
☒	☐	Clinical data

Methods

n/a	Involved in the study	
☒	☐	ChIP-seq
☒	☐	Flow cytometry
☒	☐	MRI-based neuroimaging

Antibodies

Antibodies used	Alexfluor-488-conjugated phalloidin (1:100, Life Technologies), fast myosin heavy chain marker (MHC, Abcam), Alexa Fluor-647 antibody (Life Technologies), CD31 antibody (1:100, Dako), Alexa Fluor-594 antibody (Life Technologies), endothelial-binding fluorescent isolectin GS-IB4 (Invitrogen), Human Nuclear Antigen (HNA, Millipore).
Validation	Mouse-specific antibodies were verified against cultured primary cell lines as well as tissue samples from wild-type C57 mice and human-specific antibodies were verified against commercially available primary cell lines as well as muscle tissue samples from Fisher.

Eukaryotic cell lines

Policy information about cell lines

Cell line source(s)	Mouse myoblasts (C2C12, ATCC, CRL-1772), primary human muscle precursor cells (Lonza, CC-2580) and human microvascular endothelial cells (HMEC-1, ATCC, CRL-3243), primary human microvascular endothelial cells (Lonza, CC-2810)
Authentication	Human and mouse cell lines acquired from ATCC (American Type Tissue Collection) is a major provider of cell lines to research scientists. This commercial cell line provider assess all of their lots of their cell lines by Short Tandem Repeats (STRs) which allows the distinguishing of one DNA sample from another, enabling the unique identification of cell lines. The human cells commercially purchased from Lonza are authenticated by the vendor by quality control testing completed to assure cell identity.
Mycoplasma contamination	Mycoplasma screening assays were performed using a commercially available assay on mouse myoblasts (C2C12) and

Mycoplasma contamination	microvascular endothelial cells (HMEC-1). For all primary human cells, myocoplasma testing is confirmed negative by the vendor.
Commonly misidentified lines (See ICLAC register)	n/a

Animals and other organisms

Policy information about studies involving animals; ARRIVE guidelines recommended for reporting animal research

Laboratory animals	NOD SCID mice (male, 8 weeks old, Jackson)
Wild animals	n/a
Field-collected samples	n/a
Ethics oversight	All animal studies were approved by the Institutional Animal Care and Use Committee at the Veterans Affairs Palo Alto Health Care System.

Note that full information on the approval of the study protocol must also be provided in the manuscript.
